# Machine-Learning-Assisted De Novo Design of Organic Molecules and Polymers: Opportunities and Challenges

**DOI:** 10.3390/polym12010163

**Published:** 2020-01-08

**Authors:** Guang Chen, Zhiqiang Shen, Akshay Iyer, Umar Farooq Ghumman, Shan Tang, Jinbo Bi, Wei Chen, Ying Li

**Affiliations:** 1 Department of Mechanical Engineering, University of Connecticut, Storrs, CT 06269, USA; guang.chen@uconn.edu (G.C.); zhiqiang.shen@uconn.edu (Z.S.); 2 Department of Mechanical Engineering, Northwestern University, Evanston, IL 60208, USA; akshayiyer2021@u.northwestern.edu (A.I.); UmarGhumman2018@u.northwestern.edu (U.F.G.); 3 State Key Laboratory of Structural Analysis for Industrial Equipment, Department of Engineering Mechanics, and International Research Center for Computational Mechanics, Dalian University of Technology, Dalian 116023, China; shantang@dlut.edu.cn; 4 Department of Computer Science and Engineering, University of Connecticut, Storrs, CT 06269, USA; jinbo.bi@uconn.edu; 5 Polymer Program, Institute of Materials Science, University of Connecticut, Storrs, CT 06269, USA

**Keywords:** de novo materials design, machine learning, data-driven algorithm, organic molecules, polymers, materials database

## Abstract

Organic molecules and polymers have a broad range of applications in biomedical, chemical, and materials science fields. Traditional design approaches for organic molecules and polymers are mainly experimentally-driven, guided by experience, intuition, and conceptual insights. Though they have been successfully applied to discover many important materials, these methods are facing significant challenges due to the tremendous demand of new materials and vast design space of organic molecules and polymers. Accelerated and inverse materials design is an ideal solution to these challenges. With advancements in high-throughput computation, artificial intelligence (especially machining learning, ML), and the growth of materials databases, ML-assisted materials design is emerging as a promising tool to flourish breakthroughs in many areas of materials science and engineering. To date, using ML-assisted approaches, the quantitative structure property/activity relation for material property prediction can be established more accurately and efficiently. In addition, materials design can be revolutionized and accelerated much faster than ever, through ML-enabled molecular generation and inverse molecular design. In this perspective, we review the recent progresses in ML-guided design of organic molecules and polymers, highlight several successful examples, and examine future opportunities in biomedical, chemical, and materials science fields. We further discuss the relevant challenges to solve in order to fully realize the potential of ML-assisted materials design for organic molecules and polymers. In particular, this study summarizes publicly available materials databases, feature representations for organic molecules, open-source tools for feature generation, methods for molecular generation, and ML models for prediction of material properties, which serve as a tutorial for researchers who have little experience with ML before and want to apply ML for various applications. Last but not least, it draws insights into the current limitations of ML-guided design of organic molecules and polymers. We anticipate that ML-assisted materials design for organic molecules and polymers will be the driving force in the near future, to meet the tremendous demand of new materials with tailored properties in different fields.

## 1. Introduction

Polymeric materials are ubiquitously encountered in our daily life, ranging from familiar synthetic plastics, such as polystyrene, to natural biopolymers, such as DNA and proteins. Their exceptional chemical, physical, biological and mechanical properties [1,2,3] provide the broad range of applications in biomedical, chemical, and materials science fields. A polymer is usually a long chain molecule with many covalently bonded organic molecules, or repeating units. The chemical and molecular structures of these repeating units can determine the properties of polymeric materials. Thus, the choice of a specific repeating unit grants the potential of inverse materials design. This concept of molecular design for organic molecules and polymers has been widely adopted in many fields, such as organic photovoltaics [4,5,6,7], polymer dielectrics [8,9], metal-organic frameworks (MOFs) [10,11,12,13], organic light-emitting diodes [14,15], high energetic materials [16,17,18], and design of drug-like molecules [19,20,21].

When searching and designing a new material with target properties, it is of great significance to explore the complex quantitative structure property/activity relation (QSPR/QSAR). Specifically, establishing the relationship between the molecular structure and material properties is the major task for design of organic molecules and polymers. However, due to enormous combinations of the repeating units in a polymer chain or of atoms in an organic molecule, the chemical space of polymers and organic molecules is usually extremely large. For example, the nearly-infinite space of drug-like molecules is between $10^{23}$ and $10^{60}$ [22]. Another example is the GDB-17 database, which has 166 billion molecules generated by enumeration of up to 17 atoms for an organic molecule [23]. As a result, identifying promising molecular candidates exhaustively through the chemical space is an extreme challenge if a traditional trial-and-error approach is applied, which is similar to the search of the needle in a haystack. Therefore, novel concepts of materials design and methods with effective searching capability are the keys to overcome this challenge.

The development of materials design has experienced three stages, to date. The first stage is the conventional experimentally-driven and trial-and-error materials design, guided by experience, intuition, and conceptual insights (domain knowledge). This traditional method has enjoyed success in the invention of many important drug molecules, such as penicillin [24,25]. However, this approach has unavoidable limitations. For instance, only certain macroscopic properties are available, while others can hardly be measured. Moreover, this method suffers from by-chance discovery, loss of generality, and is extremely time-, labor-, and cost-consuming. For example, it takes 13 years, on average, for discovery of a new drug molecule [26]. In the second materials design stage, thanks to the progress made in computational technologies, modeling and simulation have dominated this field for materials design. Computational methods, such as density functional theory (DFT) [27,28] and molecular dynamics (MD) [29,30], have facilitated fast materials design through high-throughput virtual screening. It is especially powerful and useful in predicting material properties when an analytic formula does not exist. For instance, in the realm of de novo drug discovery, in silico modeling to develop QSAR has been established as a plausible approach [31,32]. Yet, computer modeling still suffers from several limitations. For example, the design process is usually computationally expensive in terms of time and resources. Further, it only gives a direct mapping from a structure to its properties, while an inverse mapping from material properties to molecular structures is usually difficult to find. In the past, the inverse QSAR has been used to map a favorable region in terms of predicted activity to the corresponding molecular structures [33,34,35]. However, this is not a trivial problem: first the solutions of molecular descriptors corresponding to the region need to be resolved using the QSAR model, and these then need be mapped back to the corresponding molecular structures. The fact that the molecular descriptors chosen need to be suitable both for building a forward predictive QSAR model, as well as for translation back to molecular structure, is one of the major obstacles for this type of approach.

With the growth of materials database, as well as the development of data science and artificial intelligence (AI) in general, in particular, the invention of AlphaGo [36], we are facing a new age that has been termed the “fourth paradigm of science” [37] or the “fourth industrial revolution” [38]. This advance brings materials design into its third stage, in which data-driven methods have emerged to tackle the inverse materials design problems. In addition to experimental approaches, theoretical means, and computer simulations, data-driven materials design approaches have been considered to be the “fourth pillar” in scientific research [3]. To date, many breakthroughs and research works flourish in de novo design of organic molecules and polymers by data-driven methods. Successful frameworks include material informatics [39,40], polymer informatics [41,42], and polymer genome [43,44], to name a few. An excellent example is the development of energetic materials, which has lagged behind other materials discovery, since many of them, such as TNT and TATB, came into the market after World War II [45,46]. Using traditional methods, the most recent successful design is the invention of CL-20. However, this design case takes 30 years from its initial synthesis to be embraced in industrial application [47]. Very recently, with state-of-the-art data science techniques, the discovery of high energetic materials has been dramatically accelerated. It has been reported that, with the help of materials genome approach [48,49,50,51], a new insensitive explosive with high-energy density has been designed with much less effort [52].

Automation of organic molecules and materials design is considerably less developed than that of inorganic materials due to challenges associated with searching the vast design space defined by the almost infinite combinations of molecular constituents, microstructures and synthesis conditions [24,53,54]. Machine learning (ML), a subset of AI, has been considered as a promising method to deal with inverse molecular design. An ML-based approach can explore the underlying pattern of QSPR/QSAR in an accelerated, while efficient, manner. Because of its superior capability, the large design space can be searched exhaustively. For example, the complexity of the game Go is to the order of $10^{140}$ possible solutions [55]. Therefore, ML-assisted approaches have great potential to overcome aforementioned challenges and provide huge opportunities in materials design [56,57,58,59]. To perform an ML-assisted materials design task, acquisition of a database containing uniformly distributed data of interests is the first step, which remarkably affects the performance of the ML models to be built. The database can be obtained from public databases, published literature, or self-built by experiments or numerical simulations. In addition to the quality of the database, data representation also plays an important role in developing the ML model. The molecular structure is encoded into the feature representation as the input of ML models to establish QSAR [60]. Afterwards, the database is then divided into a training dataset and test dataset to build and validate the ML model, respectively. With the predictive ML model at hand, promising molecules can be generated through reinforcement learning (RL) or Bayesian optimization (BO).

Although there have been a few excellent review articles on this topic [58,60,61,62,63,64], this review is focused on a comprehensive and broad introduction of opportunities and challenges in the ML-guided design of organic molecules and polymers in a more friendly way to beginners. Particularly, succinct summaries of material databases, methods for feature generation, and suitable ML algorithms are provided, which can serve as a tutorial for beginners. We delineate a detailed procedure of how to use ML to predict properties of a material, and more significantly, the inverse problem of designing materials that show appropriate properties in a variety of case studies. The discussion is comprehensive, covering nearly every facet of the ML implementation and inverse design. In this review, the methods of ML-assisted property prediction and material design developed in the last decade are reviewed in a chronological order. We review ML methodologies ranging from the simplest method of linear regression to state-of-the-art deep learning (DL) methods. We believe that AI or ML will revolutionize diverse scientific fields, especially for the design of organic molecules and polymers. To embrace the potential provided by this relatively new design paradigm with less effort, this work is aimed to support material researchers who want to take advantage of ML in their own research. The paper is organized as follows. Nine typical design examples using ML-assisted approaches in various fields are presented in Section 2. In particular, to differentiate the molecular and microstructure designs, examples at either the molecular level or the microstructure level for organic photovoltaics are given. In Section 3, we reveal the open problems and challenges facing in the ML-assisted materials design and discuss possible solutions. Section 4 completes this work with a few concluding remarks. We expect ML-assisted materials design methods to play an essential role in the near future and hope that this work inspires future directions in this field.

## 2. Case Studies of ML-Assisted Materials Design

### 2.1. Molecular Design of Organic Photovoltaics (OPV)

The need for clean energy is of global importance as the consumption of traditional energy especially crude oil keeps increasing [65]. Among many clean energy techniques, design of materials to utilize solar power has been recognized as one of the most promising solutions because the huge amount of solar energy available makes it an important source of electricity. Most commercially available solar materials are crystalline silicon based. However, they suffer from a few disadvantages, like high production costs [66] and low efficiency [67]. Therefore, design of alternative materials is of great importance to better utilize solar power. The organic photovoltaic (OPV) serves as a great potential candidate due to its low cost, abundance, and installation versatility [68]. A good design example of OPVs has been developed by Aspuru–Guzik’s group. As a part of the Harvard Clean Energy Project (CEP) [69], in addition to the widely used quantum chemical computation, ML-guided high-throughput screening (HTS) was also proposed to accelerate the material discovery of a high efficiency bulk-heterojunction (BHJ) solar cell [70]. High power conversion efficiency (PCE) is the desired property that the authors tried to design for organic solar materials within the virtual chemical library.

The first step in an HTS approach is to get a pool of candidate molecules. The authors obtained a chemical library through combinatorial generation of 30 heterocyclic building blocks by either linking or fusing basic building blocks together. In this way, they finally got a library including 2.6 million conjugated molecules. To construct a prediction model to screen the generated molecular library, a dataset to build the ML model is needed. To this end, 50 molecules obtained from the literature were used as a training dataset. This dataset enclosed the molecular structures and the associated properties, which allowed them to develop a prediction model. The molecular structure information was represented by 33 physicochemical and topological descriptors calculated by the software ChemAxon [71]. Instead of mapping the molecular descriptors with the desired PCE property directly, intermediate properties, including the filler factor (FF), the short circuit current density ($J_{\mathrm{sc}}$), and the open circuit voltage ($V_{\mathrm{oc}}$), were adopted to develop the QSPR in an indirect way.

**Figure 1:**
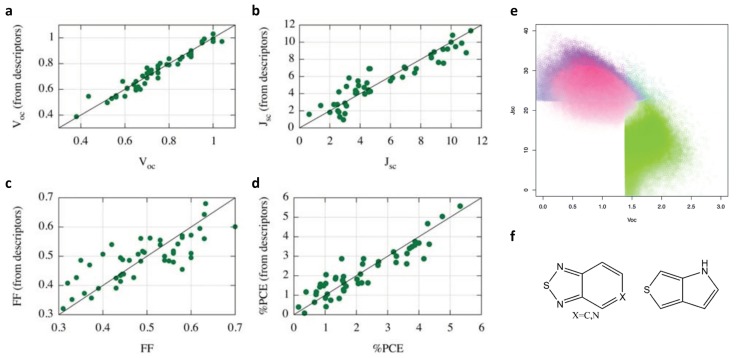
Machine learning (ML)-guided design of organic photovoltaics. (**a**–**d**) The performance of ML models on desired properties: $V_{\mathrm{oc}}$, $J_{\mathrm{sc}}$, filler factor (FF), and power conversion efficiency (%PCE); (**e**) top 10% screened molecules with highest predicted $V_{\mathrm{oc}}$ (green), $J_{\mathrm{sc}}$ (blue), and $V_{\mathrm{oc}}J_{\mathrm{sc}}$ (red); (**f**) the most promising building blocks screened by $V_{\mathrm{oc}}J_{\mathrm{sc}}$ model. The figures are adapted from Reference [70] with permission from The Royal Society of Chemistry.

The multi-linear regression model was chosen to develop the QSPR. Figure 1a–d show the prediction performance of multiple linear regression for the selected properties. The R squared values are 0.96, 0.92, 0.66, and 0.89 for $V_{\mathrm{oc}}$, $J_{\mathrm{sc}}$, FF, and %PCE, respectively. The correlations of $V_{\mathrm{oc}}$ and $J_{\mathrm{sc}}$ are great and acceptable for PCE. But the correlation of FF is poor. The authors tried other methods to mitigate this drawback by using different regression models on $V_{\mathrm{oc}}J_{\mathrm{sc}}$ and different descriptors. However, the results showed that better fit of $V_{\mathrm{oc}}$ and $J_{\mathrm{sc}}$ than FF were observed. Therefore, the ML models developed for $V_{\mathrm{oc}}$, $J_{\mathrm{sc}}$, and $V_{\mathrm{oc}}J_{\mathrm{sc}}$ were chosen to screen the virtual molecular library.

The top 10% molecules filtered out by $V_{\mathrm{oc}}$ ML model (green), $J_{\mathrm{sc}}$ ML model (blue), and $V_{\mathrm{oc}}J_{\mathrm{sc}}$ ML model (red) are given in Figure 1e. The most promising candidates are located in the upper left corner of the contour plot. Based on the $V_{\mathrm{oc}}J_{\mathrm{sc}}$ model, it is interesting to find that the building blocks presented in many desired molecules are benzothiadiazole or pyridinethiadiazole motif and thienopurrole motif (see Figure 1f). The highest predicted efficiency in the library is 10.36%, which is larger than the highest one in the training dataset of 5.32%. It further verifies that the ML-guided methods can help to accelerate the discovery of materials with excellent properties.

As an early work published in 2011, the framework proposed was innovative. However, there are several aspects to improve. First, the training dataset with just 50 cases may not be large enough to accurately figure out the underlying relationships between molecular structures and properties, compared to the large space of the virtual library to screen. Second, the obtained ML models do not have a test dataset for validation, which might not guarantee the accuracy of the ML models. Last but not least, the ML algorithm employed may be too simple to capture the complicated relation. Other ML models that are good at capturing nonlinear mapping could be tested.

### 2.2. Design of Polymer Dielectrics

**Figure 2:**
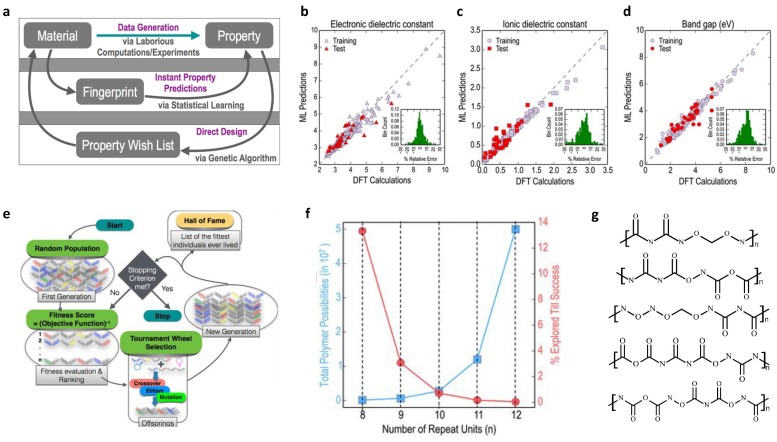
ML-guided design of dielectric polymers. (**a**) Three phases involved in this design approach; (**b**–**d**) the performance of ML model on the desired properties: electronic dielectric constant, ionic dielectric constant, and band gap; (**e**) the flow chart of genetic algorithm to identify promising candidates with desired properties; (**f**) the relation between number of building blocks and the number of possible polymers, as well as the percentage of the polymers needed to be considered; (**g**) the optimized molecular structures with 8∼12 units (C and H are not displayed explicitly). The figures are adapted from Reference [9] with permission, copyright 2016 Springer Nature.

Polymer dielectrics have been used in various applications, such as organic field-effect transistors [72], insulators [73], energy storage [74], and capacitors [44]. To meet such a high diverse demand from industry, the design of polymer dielectrics is a big challenge. With the help of ML, as well as intellectual algorithms, unprecedented progress has been made. For example, excellent work has been done by Ramprasad’s group [8,9,75]. In one of their works, state-of-the-art computational tools, ML algorithm, and genetic algorithm (GA) were combined to solve direct property prediction and inverse material design problems. Figure 2a illustrates the outline for the proposed method. Three phases are involved in this method. Data generation using first principle calculation comes in the first phase, which is followed by the construction of an ML model (second phase). After the structure–property relation is established, the third phase is the inverse design of polymers with target properties through genetic algorithm.

The training and test dataset came from first principle calculations. The candidates are chosen based on a particular chemical subspace which includes seven linearly repeated chemical building blocks (CH_2_, NH, CO, C_6_H_4_, C_4_H_2_S, CS, and O). These building blocks are commonly found for many polymer materials and can effectively represent electronic and dielectric properties of polymeric materials [76]. In order to obtain the dataset at a reasonable computational cost, four blocks forming a repeating unit was set for generating the candidate space. Furthermore, to avoid generating chemically invalid molecules, the authors pre-screened the data and 284 candidates were eventually determined.

The crystal structures of candidates were obtained by the minima hopping method [77], as well as density functional theory. The primary properties of dielectric polymers were calculated by DFT, including bandgap (*E*_gap_) and dielectric constant ($\epsilon_{\mathrm{elec}}$, $\epsilon_{\mathrm{ionic}}$, and $\epsilon_{\mathrm{total}}=\epsilon_{\mathrm{elec}}+\epsilon_{\mathrm{ionic}}$). Molecular structures of these candidates were represented by fingerprints, which can be represented by a 7×1 vector ($M_{I}$), 7×7 matrix ($M_{II}$), and 7×7×7 matrix ($M_{III}$) for individual block, block pair, and block triplet. The value in the vector or matrix denotes the occurrence frequency of corresponding building block, block pairs, or block triplet. With this representation, the chemical structure can be converted into a numerical form to build a relation between chemical structures and desired dielectric properties (high $\epsilon_{\mathrm{total}}$ and $E_{\mathrm{gap}}$) by a selected ML model.

The kernel ridge regression (KRR) with Gaussian kernel, an ML algorithm able to reproduce nonlinear relationships [78], was applied to develop a mapping from molecular structure to dielectric properties. The dataset was divided into 90% and 10% for training and test purposes, respectively. In the training step, the cross-validation scheme was also implemented to reduce over-fitting and ensure the generality of the model. Figure 2b–d show the performance of the prediction by the ML model on electric dielectric constant, ionic dielectric constant, and bandgap, compared to DFT calculations. We can tell that the prediction agrees well with the DFT calculation, which verifies the direct prediction model constructed. Moreover, the authors showed that the model developed for 4-block polymers could be applied to other repeated units with arbitrary number of building blocks. This impressive conclusion for extrapolation can guide inverse material design for polymers with longer repeating units.

To tackle the inverse design problem, the first question to answer would be how to generate the molecules. Enumeration could be an answer. However, the possible candidates grow exponentially as the block number increases, which is intractable for a case by case approach. To this end, the genetic algorithm (GA) was applied closely cooperated with the ML model. GA is an effective algorithm to find optimum candidates using the strategy imitating biological evolution, which involves crossover, mutation, and elitism [79]. In this method, a pool of candidates (initial generation) is generated randomly; for example, 300 polymers with an 8-block unit were considered by the authors. In each generation, the population undergoes different evolution operations, and a fitness score is used to evaluate the properties for an individual polymer, which is performed by the constructed KRR model. Candidates with higher fitness scores survive to the next generation (offspring). After the prescribed number of generations is reached or optimum fitness scores are obtained, the process stops. The whole procedure is explained in Figure 2e.

Figure 2f depicts the relationship between the number of building blocks and the space of total possible candidates, as well as the percentage of favorable candidates. One can see that, as the number of repeating units increases, the number of candidates increases exponentially, while the percentage of the best candidates decreases rapidly, which means enumeration and one-by-one screening is a high cost yet ineffective approach. Figure 2g shows the best structure examples given the desired properties (target $E_{\mathrm{gap}}=5\mathrm{eV}$ and $\varepsilon_{\mathrm{total}}=5$), with the number of building blocks ranging from 8 to 12.

### 2.3. Molecular Design of Organic Light-Emitting Diodes

**Figure 3:**
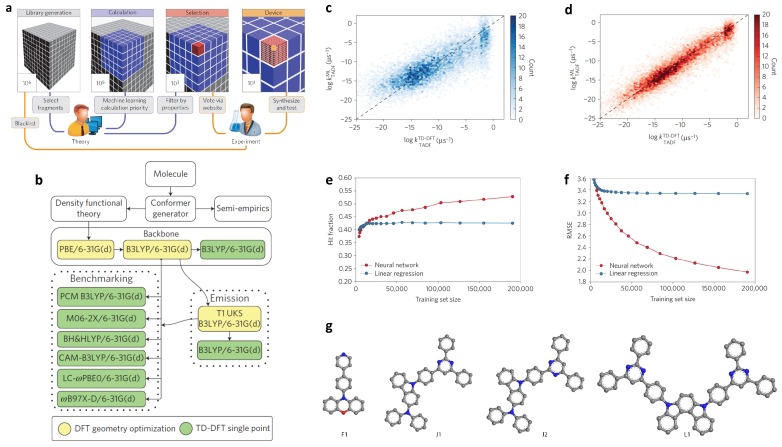
Integrated design of organic light-emitting diodes (OLEDs). (**a**) Schematic of the integrated design method; (**b**) flow chart of quantum chemical computation; (**c**,**d**) the coefficient of determinant for linear regression (0.80) and neural network (0.94); (**e**,**f**) the relation between hit fraction and root mean square error (RMSE) with respect to the training set size; (**a**–**f**) are adapted from Reference [83] with permission, copyright 2016 Springer Nature; (**g**) the best candidate molecular structures (gray, blue, and red nodes) denote carbon, nitrogen, and oxygen atoms, respectively.

Organic light-emitting diodes (OLEDs) have great potential to be applied in the third-generation display devices. OLEDs have promising properties, including energy-saving and long-lasting working time [80], both of which outperform liquid crystal display (LCD) technology. Traditional fluorescent emitters allow only singlet-singlet transition, resulting in the limited harvest efficiency. Even though phosphorescent OLEDs have higher harvest efficiency, their application is restricted by high costs since a necessary ingredient (iridium) is very expensive. The thermally-activated delayed fluorescent (TADF) technique has been proposed to mitigate these limitations [81,82]. Using this method, excellent work has been done. For example, Aspuru-Guzik et al. reported that experimentally tested external quantum efficiency (EQE) were up to 22% [83]. In their work, the authors combined the quantum chemical computation, cheminformatics [84,85], machine learning, industrial expertise, synthesis, and testing to construct an effective design framework for the design of blue TADF OLEDs emitters. Figure 3a illustrates the schematic of the framework.

Utilizing their in-house code based on the RDKit package [86], a virtual chemical library of OLED molecular candidates was generated as a starting point. The generation of the library was directed by chemical intuition, quantum simulation, and experimental work. To increase the pool of the library, the authors began with a group of fragments and used combinatorial enumeration with constraints. The imposed constraints include the following considerations: the structure requirement of the TADF OLEDs molecules to be in the form of donor-(bridge)x-acceptor (where *x* can be 0, 1 or 2 ), the symmetry in molecular substitution, molecular size, vapor processing, optical properties, and synthesis accessibility. Furthermore, a blacklist of unwanted structures, such as chemically unstable molecules, was compiled to further constrain the growth of the library. Following these rules, 110 donors, 105 acceptors, and seven bridges came out. The library finally grew into a big space with 1.6 million candidate molecules.

Direct screening of this library by quantum chemical simulation alone is intractable. Therefore, 40,000 candidates were randomly selected and evaluated by time-dependent DFT (TD-DFT). The flow chart of the calculation is shown in Figure 3b. Since the nonradiative decay rates are difficult to predict by theoretical or numerical approaches, the delayed fluorescent rate constant $k_{\mathrm{TADF}}$ is selected as the desired property, which was estimated through its relation to singlet-triplet gap $\Delta E_{ST}$ and oscillator strength *f* [83]:

(1) $$k_{\mathrm{TADF}}=\alpha \frac{f}{1+3\exp(\Delta E_{st}/kT)},$$

where α is material constant, *k* the Boltzmann constant, and *T* the absolute temperature. The two parameters $\Delta E_{ST}$ and *f* were obtained through DFT quantum chemical calculation on the candidate molecules. In this fashion, the property ($k_{\mathrm{TADF}}$) of candidate molecules can be evaluated by DFT calculations. As a result, the training dataset enclosing molecular structures and respective properties is ready to build a mapping between them using ML.

To construct an ML model, the molecular structure was first represented in a simplified molecular-input line-entry system (SMILES) form [87], and then was converted to a vector with fixed length using extended connectivity fingerprints (ECFP) [88]. Note that the SMILES is a specification in the form of a line notation for describing the structure of chemical species using short ASCII strings. Then the neural network (NN) with two hidden layers was selected as the ML model, which was trained to map a molecular ECFP to its property $k_{\mathrm{TADF}}$. The training objective was to minimize the root mean square error (RMSE) of predicted $\log(k_{\mathrm{TADF}})$.

After this ML model was established, it was then employed to further screen the rest of the library. The candidates that gave the best predictions were moved up for TD-DFT calculation. In the meantime, the neural network model was retrained as new data were added, which further increased the predictive accuracy of the ML model. Prediction by linear regression was also carried out for comparison. It is easy to see from Figure 3c,d that the coefficient of determination ($R^{2}$) of the test dataset were 0.80 and 0.94 for linear regression and neural network algorithm, respectively. Compared to the linear regression, the neural-network-based ML model performs better with a large dataset. It is also found that the performance of NN model is more dependent on the training data size (Figure 3e,f); while with larger data size, the performance improvement of LR model is imperceptible.

When the library was screened to a human-tractable range, a collaborative decision-making procedure was conducted by two to six synthetic organic chemists to evaluate the molecules. Four optimum molecules (Figure 3g) were then synthesized and tested to validate the prediction by the ML model. They showed that the EQE is over 22%. It was proved that an ML-guided materials design approach for OLEDs can be really powerful.

### 2.4. Design of Polymeric Solar Cell

Polymeric materials are widely applied as active photoabsorbers in most organic solar cells. The donor-acceptor type of polymers is a prospective candidate for design of solar cells. By tuning the donor and acceptor units, the highest occupied molecular orbital (HOMO) and the lowest unoccupied molecular orbital (LUMO) can be designed [89]. In order to capture sufficient solar energy, the ideal optical gap is to fall in the range of 1.1–1.7 eV [89]. Due to the large number of possible donor-acceptor combinations, it is suitable to use ML models to find promising solar cells. A recent work by Jorgensen et al. gives an excellent design example. In their work, they not only obtained direct property prediction models but also realized inverse materials design with desired properties by the so-called grammar variational autoencoder (GrammarVAE) [89]. Among many of the important properties of polymeric solar cells, LUMO ($\epsilon_{\mathrm{LUMO}}$) and the lowest optical transition energy ($\epsilon_{\mathrm{opt}}$) were adopted by the authors as the design targets.

**Figure 4:**
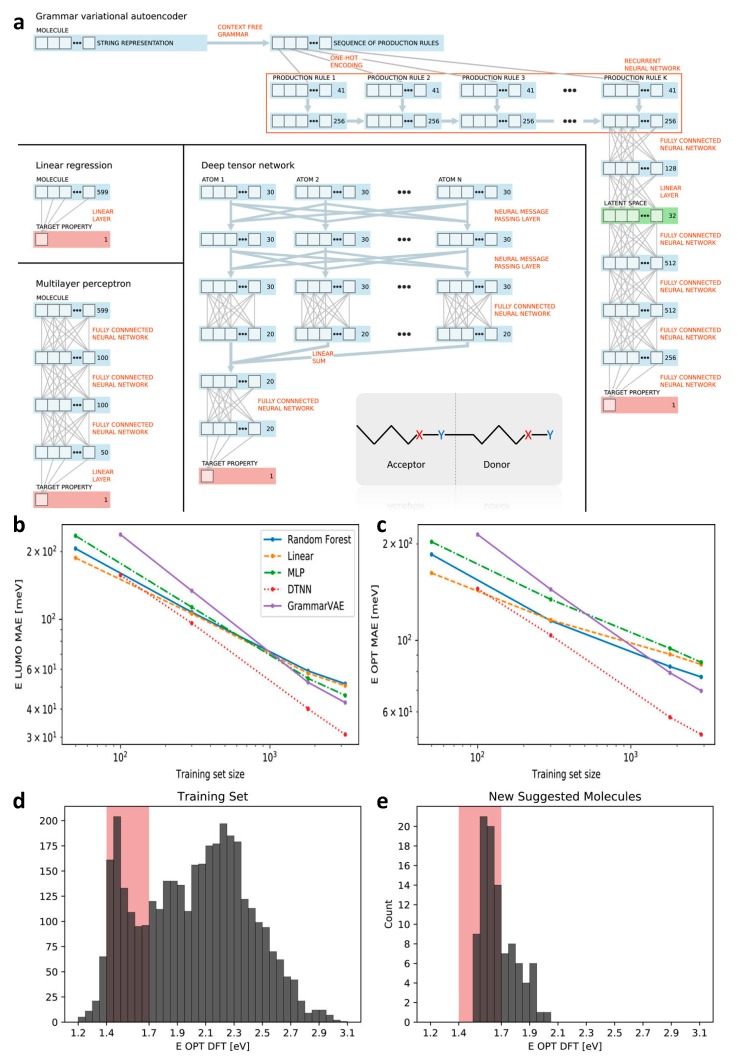
Design framework of polymeric solar cell. (**a**) Data flow of four different ML models (the gray shaded box in the bottom of it is a representation of donor-acceptor structure with X and Y the side groups, the number of which are variable); (**b**,**c**) performance comparison of different ML models; (**d**,**e**) molecules distribution in training dataset and new suggested molecules for $\epsilon_{\mathrm{opt}}$ (shaded area stands for target property range). These figures are adapted from Reference [89] with permission, copyright 2018 AIP publishing. DFT = density functional theory.

The figure in the shaded box in Figure 4a illustrates a typical organic solar cell molecule considered by the authors. To build a virtual library of possible molecules, 13 acceptor, 10 donor moieties, 9 possible side-chains, and atomic substitutions were used. Combinatorial enumeration of the basic elements resulted in $10^{14}$ monomer structures. The authors first pre-screened the library by ignoring the count number differences, ignoring relative positions of side groups, and pre-optimizing the structures. A virtual library containing 3938 monomers was then constructed. DFT calculations were conducted to get the corresponding properties to form a complete dataset.

Representation of molecules is one important element in the development of an ML-based materials design approach. The commonly utilized approaches for molecular descriptors include: Coulomb matrix [90,91], bag-of-bonds [91,92], and molecular graphs [61,63,93]. However, the authors argued that the spatial information of molecules utilized by these representations might not be available [89]. SMILES string is another commonly used molecular structure representation. Generative ML models, for example, the variational autoencoder (VAE), are usually applied to generate molecular SMILES to get a chemical library [24,54,63]. But this method still suffers from generating chemically invalid molecules. To alleviate the problems, the authors integrated a simple string featurization and GrammarVAE. By imposing chemical grammar at the decoding phase, they can prohibit generating syntactically invalid molecules.

Three different representation methods were employed for comparison, including fixed length vector representation, string representation, and XYZ-coordinate. The former two representations do not need the spatial configuration of the molecular structure, while the third representation requires spatial configurations obtained by DFT calculation. Though computationally costly, the authors intended to show how the spatial information could influence the accuracy of ML algorithms. Five types of ML models were selected to construct the structure–property relations, namely linear ridge regression (LRR), multi-layer perceptron (MLP, three hidden layers with 100, 100, and 50 units, respectively), random forest regression (RFR), the deep tensor neural network (DTNN), and the grammar variational autoencoder (GrammarVAE). To distinguish the performances of these different ML algorithms, various combinations of molecular representations and ML models were carried out. Specifically, LRR, MLP, and RFR used fixed length vector representation; DTNN adopted the XYZ-coordinates; and GrammarVAE applied the simple string representation. The schematic of the data flow in each ML model is shown in Figure 4a.

The performances of these models are compared in Figure 4b,c. They show the performances of all the models increase as the data size increases. They also show that DTNN model behaves better than the other. The better performance of DTNNA is considered to be a result of utilizing spatial information as input. In addition, we can see GrammarVAE gives the lowest mean average error when a large dataset is employed for model development.

GrammarVAE was then used to inversely design promising candidates with desired properties. All the data was used as a training dataset to generate SMILES strings. BO method [54] and approximated calculation of conditional probability were applied to assist generation of molecules. The top 100 candidates were selected and evaluated by DFT calculations of $\epsilon_{\mathrm{opt}}$, which are shown in Figure 4d,e. The results show that the percentage of promising candidates increased from 11% (random distribution of the molecules in a wide property range) to 61% in the screened dataset (the molecules distribute close to the desired property range). It confirms that ML-based algorithms can accelerate the process of materials discovery with desired optical properties.

### 2.5. Design of High Energetic Materials

The development of energetic materials with high energy density yet low sensitivity to external stimulus has great challenges. The trade-off between high energy density and low sensitivity brings with many difficulties when designing energetic materials [52]. In addition, the limited number of available databases restricts the application of an ML-assisted design method. The data scarcity of energetic materials originates from two aspects: (1) experiments are inherently dangerous; and (2) quantum chemical calculations are prohibitively expensive. Therefore, the major challenge in ML-assisted design of energetic materials is how to develop a sound model with limited data. Recent works by Chung et al. demonstrated that good performance could still be achieved by using only 109 molecular data points [91,94]. In these works, several featurizations and various ML models were combined for dual study objectives: fast prediction of detonation properties (explosive energy, detonation pressure, and velocity) and comparison of the molecular representations and ML models.

The dataset of 109 candidates in their studies came from the literature [95], which is uniformly distributed in ten distinct compound classes and, more importantly, verified by experiments. DFT calculation and analytical approach were utilized to obtain the properties of these candidates. Nine different properties are considered, such as density, heat of formation of the solid, TNT equivalent per cubic centimeter, etc. The authors indicated that with a diverse training dataset, the model trained should be relatively generative.

To establish a reasonable ML model, it is required that the number of molecular features should be much less than the total number of molecules in the dataset. As a result, featurization is of great importance. Popular representation methods, such as SMILES, may not be suitable since these representation methods need a large dataset to train the ML model. The authors suggested that chemical intuition and domain knowledge can assist feature selection. In one earlier work [91], they chose five featurizations: custom descriptor set (CDS, a vector including 21 customized parameters, like raw counts of carbon and nitrogen), sum over bond (SoB, a vector that contains how many different bonds are presented), Coulomb matrix (CM, coordinates and nuclear charges were transformed to the Coulomb matrix eigenvalue spectra representation, which is invariant of the transformation and rotation of the molecular structure), bag of bonds (BoB, a bag containing the number of occurrence of different bonds), and fingerprinting (it transfers molecular graphs into vector form).The kernel ridge regression (KRR), ridge regression (RR), support vector regression (SVR), random forest (RF), and k-nearest neighbors (KNN) were selected as the ML models. In a later work [94], they also adopted LASSO regression, Gaussian process regression (GPR), and neural network (NN) to develop prediction models using the same dataset, plus additional 309 molecules from another reference [96].

**Figure 5:**
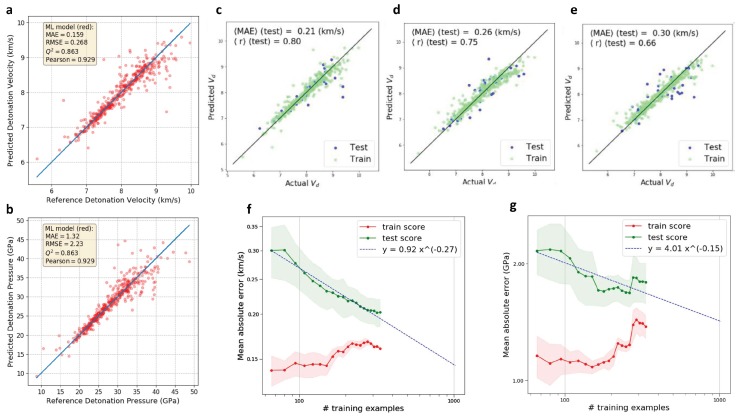
Detonation property prediction of energetic materials. (**a**,**b**) The performance of the neural network model for prediction of detonation velocity and pressure; (**c**–**e**) prediction accuracy of LASSO, Gaussian process regression (GPR), and neural network (NN), respectively; (**f**,**g**) left: learning curves of ML model for detonation energy; right: detonation pressure. (**a**–**e**) are adapted from Reference [94] with permission. (**f**,**g**) are adapted from Reference [91] with permission, copyright 2018 Springer Nature.

They showed that the SoB featurization always performed the best compared to the rest in prediction of detonation properties. Among all of these ML models, KRR and RR outperformed SVR, RF, and KNN; GPR and NN outmatched LASSO regression. The performance of the neural network model for the predictions of detonation velocity and pressure are shown in Figure 5a,b. The accuracy of ML predictions by LASSO, GPR, and NN are demonstrated in Figure 5c–e, respectively. Good performances are observed for the ML models applied. Furthermore, to study the influence of data diversity on the ML model, the authors adopted another small dataset including 25 molecules (narrow and in the same molecular class) [97] to construct a prediction model. They found that the ML model developed was not generative beyond this specific class of molecules since it did not capture the difference between various classes. Thus, when predicting properties of molecules from other classes, the model performance is poor. They emphasized the importance of the applicability domain of ML models. The learning curves of data dependent modeling have also been investigated, which are shown in Figure 5f,g. We can see that, as the data size increases, the gaps between the training and test curves decrease.

### 2.6. Design of Polyimides with High Refractive Index (RI)

**Figure 6:**
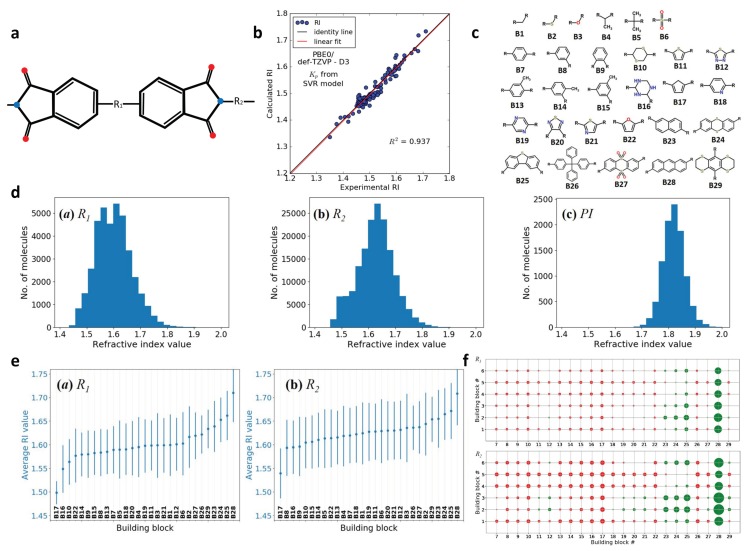
Accelerated design of polyimides (PIs) with high refractive index. (**a**) The core structure of PI with R1 and R2 group (blue and red nodes denote nitrogen and oxygen atoms, respectively); (**b**) the performance of the support vector regression (SVR) model (adapted from Reference [102] with permission, copyright 2018 AIP publishing); (**c**) 29 building blocks; (**d**) the distribution of molecules versus the RI values for R1, R2, and PIs; (**e**) RI values in terms of each building block for R1 and R2; (**f**) Z-score of building pairs for R1 and R2 (**c**–**f**) are adapted from Reference [100] with permission, copyright 2019 American Chemical Society.

Polyimides (PIs) are potential materials for the next generation optic and optoelectronic applications. To achieve superior performances, the desired values of refractive index (RI) should be larger than 1.7 [98]. However, the typical RI values of many current PIs are between 1.3∼1.5. Therefore, it is of great significance to design new PIs from the molecular or monomer level with desired RI values. A monomer structure of PIs is determined by two basic blocks called R1 and R2 [99], as shown in Figure 6a. The properties of PIs can be tuned by controlling these two basic blocks [100]. In the design of basic blocks, there are still problems to solve. First, the chemical space is huge because there is large amount of possible combinations of the basic blocks. Second, screening all the candidates by traditional methods is cumbersome and not cost-effective. Recent publications have showed that utilization of ML algorithm and first principle calculations can accelerate the discovery of promising PIs [100].

Different from the aforementioned examples, the work did not construct an ML model by directly mapping a PI’s molecular structure to the RI values. Instead, they tried to build a relationship between the molecular structures of PIs and two intermediate properties (the polarizability α and the number density *N*). These two intermediate properties are related with RI values through analytical analysis. An analytical equation between α, *N* and RI values are given as follows:

(2) $$n_{r}=\sqrt{\frac{1+2\alpha N/3\epsilon_{0}}{1-\alpha N/3\epsilon_{0}}},$$

where $n_{r}$ is the RI value, and $\epsilon_{0}$ is the material permittivity in vacuum. The values of polarizability α were obtained from first principle calculations. The number density *N* was obtained by applying data modeling via the van der Waals volume $V_{\mathrm{dWv}}$ and packing fraction $K_{p}$ ($N=K_{p}/V_{\mathrm{dWv}}$). In addition, $V_{\mathrm{dWv}}$ was calculated by Slonimskii’s method [101], and $K_{p}$ was predicted by support vector regression (SVR). As a result, given a molecular structure, the RI value can be predicted by using this protocol with integrated first principle calculations and ML models.

To develop an ML model, a dataset of 84 polymers from literature was selected, in which the $K_{p}$ was obtained by experiments. The number of monomer units was used for the structure feature, and $K_{p}$ value was the property to be related to. All the data was treated as a training dataset to build an SVR model. To validate the ML model, an external dataset including 112 non-conjugate polymers was selected as the test dataset. The performance of the SVR model is shown in Figure 6b. As we can see, a good correlation has been found with $R^{2}=0.94$. Thus, the ML model is acceptable for $K_{p}$ prediction.

Once the prediction model is constructed, it is then employed for the high-throughput screening to find promising candidates. To this end, a molecular library was determined first. A group of 29 building blocks, including 6 linkers (B1–B6) and 23 aromatic moieties (B7–B29), serves as the base to generate the library (see Figure 6c). Following a combinatorial approach, the authors found 38,619 candidates for R1 and 171,172 candidates for R2, resulting in a chemical space including 6.6 billion candidates for PIs. Instead of searching the whole chemical space, they narrowed down the candidate pool by only selecting the top 100 R1 and 100 R2 candidates with highest RI values. In this way, the search space was reduced to just 10,000 candidates.

The distributions of RI values with respect to the R1 group, R2 group, and the combined PI structures are shown as Figure 6d. As we can see, the authors found promising candidates with RI values larger than 1.7. In the meantime, they also tested the influence of individual block and block pairs on the RI value through the Z-score analysis. With the help of Z-score analysis, the correlations of each building block and block pairs to the RI values are quantified. This sensitivity analysis can distinguish contributions from block or block pairs, so that the key group can be located. From Figure 6e, we can tell that R1 and R2 candidates containing B24, B25, and B28 have higher RI values than the rests. Figure 6f suggests that block pairs containing B28 give the best RI performance. In a nutshell, the building block B28 seems to be the most promising one. It inspires us that a data mining technique can be applied to find underlying patterns between structures and properties, which can help reduce dimensionality and select the main features among many.

### 2.7. Polymers with High Thermal Conductivity

**Figure 7:**
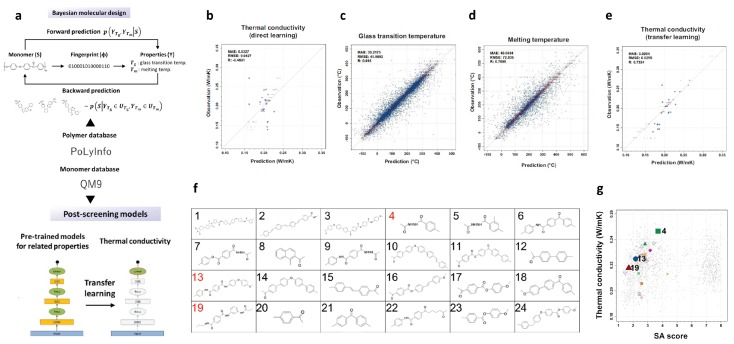
Integrated design for polymers with high thermal conductivity. (**a**) The proposed ML approach for materials discovery; (**b**) the performance of a direct learning algorithm; (**c**,**d**) validations of the trained linear regression model for glass transition temperature and melt temperature, respectively; (**e**) validation of the transfer learning; (**f**) the screened molecular candidates, in which the number are synthesized in red color; (**g**) validation of the synthesized molecules. The figures are adapted from Reference [106] with permission, copyright 2019 Springer Nature.

With the rapid development of size and integration density in electronic devices, the massive heat generated poses challenges on the performance and lifespan of these devices. Thus, the discovery of heat dissipating polymeric materials with high thermal conductivity is of great importance to maintain reliable performance, as well as a long lifetime, for these electronic devices [103,104,105]. However, in the design of polymeric materials, an ML model to directly link a structure and thermal conductivity is difficult to construct. It is because common public databases including thermal conductivity are limited. Moreover, due to the massive chemical space, building a large database by molecular dynamics is currently restricted by the extremely long computation time. Nonetheless, a recent work gave a good idea for how to deal with this design problem. In this work, the authors incorporated ML algorithm, expertise from material synthesis, and advanced measurement technology [106]. The synthesized polymers were reported to have thermal conductivities of 0.18–0.41 W/mK, which match the cutting-edge ones in thermoplastics without any fillers. Figure 7a shows the schematic of the ML-guided design process, which consists of a forward prediction step, a Bayesian molecular design step, and a backward prediction step.

In the first and forward prediction step, the data was obtained from the public database PoLyInfo [107] and QM9 [90,108]. There were a hundred properties recorded in accordance with constitutional repeating units. However, the data for thermal conductivity had only 28 instances, which limited the performance of a predictive ML model. Additionally, as a second order tensor, thermal conductivity is sensitive to polymer processing. As a result, the direct structure-property relation is less convincing. As indicated in Figure 7b, the coefficient of determination is only −0.4601, which is very bad. Alternatively, an indirect approach was adopted by the authors for the QSPR development. Based on heat conduction theory and reported literature [109,110], they first mapped structures of molecules to proxy properties, including glass transition temperature $T_{g}$, melt temperature $T_{m}$, density ρ, and heat capacity $C_{V}$. A transfer learning process was then applied to link the structure to the desired thermal conductivity property. More importantly, the data of the surrogate properties was adequate to obtain a reasonable model. For instance, PoLyInfo recorded 5917 and 3234 structures for $T_{g}$ and $T_{m}$, respectively.

Molecular fingerprints (ECFP) was selected as the molecular feature. A linear regression model was trained based on 80% of the dataset. As illustrated in Figure 7c,d, the models gave good predictions for glass transition temperature and melt temperature. By using different training data, 1000 pre-trained models were constructed, in which the weight parameters involved were refined using the limited data of thermal conductivity in the library. From this model pool, the best transferable model to predict thermal conductivity was identified. Following these approaches, a transfer learning model was constructed to correlate thermal conductivity to the molecular structure. However, the performance of the model is unreliable since the test dataset only has 28 data points. Therefore, in the molecular design step, the intermediate properties $T_{g}$ and $T_{m}$ were adopted as design targets, while the transferred model was employed as a post-screening tool for accelerated molecular generation. Figure 7e verified the transfer learning model with certain precision.

The next step was the generation of a molecular library. A successful dataset with high percentage of chemical valid structures is the key for molecular design. To avoid the generation of a large amount of invalid structures by using chemical fragments and their combinations, the so-called Bayesian molecular design method was carried out [111]. The Bayesian method requires significantly less data for model training compared to the recurrent neural network (RNN) [112] and variational autoencoder (VAE) [113]. Incorporating the SMILES representation and Bayesian ML model, the authors used the self-developed n-gram technique for molecular generation. In the molecular generation process, constraints were considered, including synthetic accessibility (SA), ease of processing, validity of chemical bond, chemical stability, and liquid-crystalline polymers (LCPs) likeness. Finally, 1000 candidates were generated.

The third step was to screen these 1000 candidates. The authors considered three key factors, namely LCP-like structures, higher SA score, and ease of processing (which required $T_{g}\leq 300$ °C). As a consequence, 24 candidates were identified for further investigation (see Figure 7f). Three promising candidates among these 24 candidates were further synthesized and tested. The comparison between the prediction and experimental results of the screened structures is showed in Figure 7g. We can see that the screened candidates have desired properties, which verifies the proposed protocols.

### 2.8. De Novo Drug-Like Molecular Design

Thanks to the recent advances of computer science techniques, various ML models have been introduced into drug discovery, for instance, the recurrent neural networks (RNNs), the generative adversarial network (GANs), and variational autoencoder (VAE). Many previous works [54,112,114,115] focused these models on screening of chemical libraries, or they may not be able to design drug-like molecules by well controlling the properties in a specific range [116]. On the other hand, Popova et al. recently reported that, using deep reinforcement learning (RL) algorithm, they were able to bias the generation of molecular library towards specific range of chemical, physical, and/or biological properties [116].

**Figure 8:**
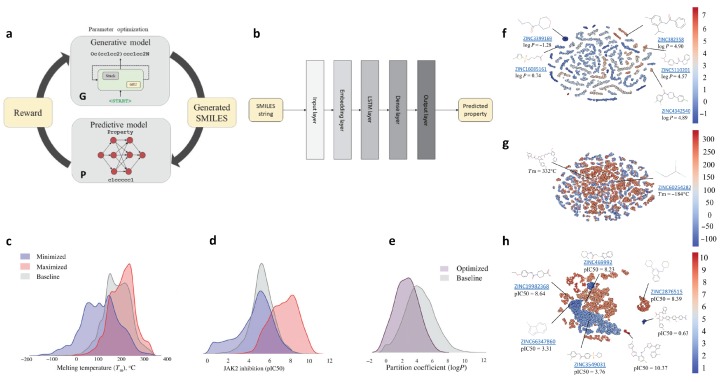
De novo drug-like molecular design framework. (**a**) The proposed framework of reinforcement learning (RL) model; (**b**) flow chart of the predictive model; (**c**–**e**) properties distributions of RL model versus baseline model (no RL); (**f**,**g**,**h**) clustering of generated molecules. The figures are adapted from Reference [116] with permission, copyright 2018 AAAS.

In their work, two deep neural networks, along with generative and predictive models, were developed independently. These two models were then combined into a RL framework, as shown in Figure 8a. There are two phases in the proposed framework. In the first phase, the generative model and predictive model are trained separately. In the second phase, they are trained together with RL to generate new molecules with targeted properties. The generative model aims to learn the underlying pattern between a molecule and its SMILES string so that it can generate novel chemically feasible candidates. The predictive model (Figure 8b) is designed to evaluate the candidates generated by the generative model. In the RL step, the predictive model assigns rewards if the desired candidates are generated, otherwise assigns penalties to the given candidates. In this way, the generation of new molecules is biased towards the region with target properties.

In developing the generative neural network, the database adopted for training is obtained from the ChEMBL21 database (https://www.ebi.ac.uk/chembl/). The deep network of this model had a Gated Recurrent Unit (GRU) layer with 1500 units and a stack augmentation layer with 512 units. In order to generate chemically legitimate molecules (correct valence, balance of ring opening and closure, or bracket sequences with different brackets styles) in SMILES form (the output of the model), the authors used the stack memory augmented network to learn the underlying grammar towards chemical feasible strings. The model was then trained with about 1.5 million structures from the ChEMBL21 database to learn the SMILES grammar of real chemical structures. To validate the model, they generated 1 million molecules. The validation checker by using ChemAxon [71] showed that 95 percent of the generated compounds were valid and chemically sensible. It was also shown that less than 0.1% of the generated molecules was from the training dataset. It indicates that the model did learn the grammar instead of memorizing the training dataset. Moreover, the synthetic accessibility (SA) score calculation showed that most generated molecules were synthetically accessible (99.5% molecules have SA score below 6 above which molecules are considered not easy to be synthesized). By comparison, the same ML model without the stack memory showed different results in two aspects: (1) the chemically valid percentage was reduced to 86%; (2) the number of similar molecules to the training dataset was increased. All of these aspects justify the importance of using stack memory for learning.

When developing the predictive neural network, $T_{m}$, logP, and ${\mathrm{pIC}}_{50}$ for JAK2 were selected as the target properties. SMILES strings were selected as the only molecular representation. The deep network employed had three layers: a long short-term memory (LSTM) layer with 100 neurons and tanh activation function, a dense layer with 100 neurons and Rectified Linear Unit (ReLU) activation function, and the output layer with one neuron and identity activation function. 5-fold cross-validation technique was adopted to build the predictive ML model. The model was then applied to external prediction for model validation. Good predictive performance was observed. For example, the prediction gave a $R^{2}=0.91$ for n−octanol/water partition coefficient (logP). In addition, the prediction for melting temperature ($T_{m}$) was shown to be comparable to the prediction by state-of-the-art descriptor based random forest model [117].

Incorporated with the generative and predictive models, the RL was then formed to design molecules with controllable physical/chemical/biological properties ($T_{m}$, logP, and JAK2 inhibition). To design target properties with maximum or minimum range, the authors gave high rewards to molecules with more benzene rings, and more small group substitutes (like −NH_2_,−OH). On the other hand, they penalized molecules with undesired structures like bromine or carboxyl group. Figure 8c–e demonstrated the property distributions of training dataset and generated library (10,000 molecules). One can see that the generated molecules shifted the properties from the baseline to maximum or minimum range, which verified the model constructed. To further visualize the generated molecules, the t-distributed stochastic neighbor embedding for dimensionality reduction was carried out as shown in Figure 8f–h. In these figures, a point refers to a molecule and is colored by its property value. We can see that there are clusters for logP and JAK2 inhibition, while no cluster for $T_{m}$, which can provide useful information for those molecules.

### 2.9. Microstructure Design of Organic Photovoltaic Solar Cells (OPVCs)

The materials design approaches presented in previous cases were attempted at only molecular levels, while the current example zooms out to the nanoscale level. In particular, these previous cases mainly focus on the chemistry–property relationship for organic molecules and polymers, while the processing–microstructure–morphology–performance relationship also plays an important role in the design of advanced functional materials and devices [118,119,120,121,122]. Different from the OPV example given in Section 2.1, where the focus was more on chemical composition and resulting molecular structure, another approach identified is the optimization of the microstructure for OPVCs to improve the performance (which in this case is called Incident Photon to Converted Electron-IPCE efficiency) [120]. It is because the ultimate performance of the devised devices is highly dependent on the material processing and the microstructure. The processing-structure-performance (PSP) relationship [123] is the key to design materials with targeted performances. As such, a case study on microstructure design of OPVCs is presented to make this study more comprehensive.

The quest for high performance metamaterials by cost-effective fabrication techniques has put design of nanostructures material systems (NMSs) [124] in the limelight. Compared to the costly top-down fabrication techniques for periodic NMSs, the quasi-random nanostructures can be fabricated by low cost, bottom-up, processes [125]. One such example of quasi-random nanostructures is OPVCs [6,126,127]. In order to get optimal performance, multiple structure and processing parameters, such as thickness of active layer, donor-acceptor ratio, annealing temperature, etc., need to be optimized simultaneously. There have been multiple attempts to optimize the performance by changing one or two parameters at a time, but a more recent approach provides a framework which can take more parameters in an efficient manner to reach the optimal structure [128].

**Figure 9:**
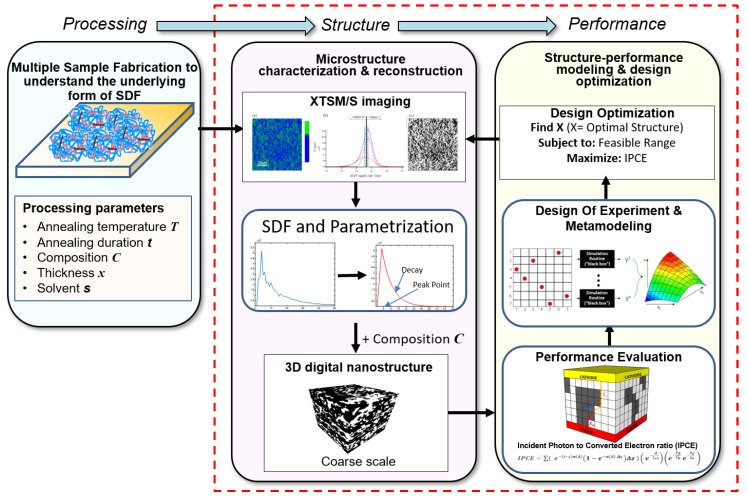
A framework for designing active layer of organic photovoltaic solar cells (OPVCs) via spectral density function. This figure is adapted with permission from Reference [128], copyright 2018 American Society of Mechanical Engineering. SDF = spectral density function.

Although the authors propose to complete the PSP chain, they focus their work on the first stage, i.e., to establish the Structure-Performance (S-P) relationship (enclosed in red dotted box in Figure 9). Because of the high dimensionality of the underlying structure, it becomes necessary to reduce the dimensionality by extracting only the salient and potentially useful features of the microstructure. Microstructure characterization and reconstruction (MCR) [119,129] provides a quantitative approach to analyze microstructure, while reducing the dimensionality. Among the many MCR techniques [119,129,130,131,132], the authors chose spectral density function (SDF) [118,133,134,135] because of its proven efficacy to represent [118,133,134] and design [134] quasi-random NMSs, and also because of its physical association with processing conditions [118]. SDF is a one-dimensional function of spatial frequency, calculated as the radial average of the squared magnitude of Fourier spectrum of a quasi-random structure [135,136] and represents the structural correlation in Fourier space. To make the design problem more efficient, this one-dimensional function can be further reduced to a couple of variables by parameterizing the SDF curve. For this study, two parameters were selected, i.e., Decay and Peak Point, to represent SDF.

After characterization, a statistically equivalent 3D microstructure was created using SDF. A novel, physics-based performance evaluation strategy was developed in this work to evaluate the efficiency of this reconstruction. In the next step, a performance optimization problem was setup to determine the optimal microstructure. Every 3D digital reconstruction required significant computational time. To save the computational cost of reconstruction and make the design more efficient, ML technique was brought in. Metamodel, which is a popular category in ML, allows the usage of a few intelligently selected datapoints to estimate the original model. This metamodel can then be used to find the global/local minimum. The metamodel in this study was based on the kriging technique [137] and Optimal Latin Hyper Sampling [138] technique was used to find the data points. For three input variables (two SDF parameters, and Volume Fraction), 45 data points were reconstructed and evaluated to fill the design space to build the metamodel. The range for SDF parameters was based on a couple of SDFs from X/STM images of OPVC samples, and the range for Volume Fraction was selected based on the literature studies. In the final step, Sobol sensitivity analysis [139] was also carried out to extract most important variables.

The study showed a 36.75% increase in the IPCE value after structural optimization. Upon investigation, it was also concluded that the PCBM (Phenyl-C61-butyric acid methyl ester) volume fraction (or composition) was the most influential design variable followed by Decay (one of the SDF parameters). The current work lacks experimental validation, so the next logical step for this study could be to complete the PSP chain and verify the optimized result against physical experiments.

## 3. Discussion

The aforementioned nine examples about organic molecule and polymer design demonstrate the huge potential for applying ML to accelerate the materials design process in various fields, as summarized in Table 1. However, challenges still exist in application of ML-guided materials design approach. In what follows, details about the problems associated with the ML approach will be discussed. In particular, materials database, feature selection and extraction, and ML models for molecular generation and inverse materials design will be further discussed.

### 3.1. Materials Database

The acquisition of a large materials database is the first step and the foundation to develop an ML model. Within the database, material structures and corresponding properties are enclosed, which could be obtained either from experimental works or numerical simulations. The characteristics of the database strongly influence the capability of the ultimate ML model since the model is trained and validated by the database. These characteristics include adequate size, diversity, and uniformity across the chemical space [140]. Diversity and uniformity are even more important to construct a predictive ML model for interpolation, extrapolation, and exploration. Even with a small database, effective ML models could still be built with reasonable accuracy, as shown in the energetic materials design case [91,94] and polymer thermal conductivity design case [106].

Considering the tremendous effort needed to establish a database from scratch, a better way is to use existing public databases, provided that the structures and properties of interests are available. Some popular materials databases are listed in Table 2 [57,60]. If public databases are not available in some cases, natural language processing (NLP) can be applied to extract data from published literature. For example, Cooper et al. [141] adopted the text-mining tool ChemDataExtractor [142] to construct a database of 9431 dye candidates in their polymeric solar cell study. Their database constructed through NLP contains the information of chemical structures, maximum absorption wavelengths, and molar extinction coefficients. Based on this database, the authors then identified a promising candidate from the molecular library by high-throughput screening, which was further verified by experiments. The experimental test showed that it demonstrated comparable PCE to the organometallic dye N719. The solar cell study successfully shows the effectivity of using NLP for data mining in materials science fields.

### 3.2. Machine Learning Model

#### 3.2.1. Feature Selection and Extraction

With the obtained database at hand, the next key step is to transform a material’s structure into a digital coded format that can be used as the input for ML models. However, there are no fixed rules for feature selection. In general, representations of chemical structures are expected to meet several requirements. First of all, the representation should be based on chemical or physical knowledge. It is shown that representations embedding the physics and structural information help ML models perform better [60,61]. Additionally, the representation of a certain chemical structure should not change under transformations, such as spatial translation and rotation [143]. Moreover, it is better for the representation methods to be unique and invertible [60]. Generally, molecular representations can be classified into three types: discrete, continuous, and weighted graphs [24]. Several commonly used representations are categorized in Table 3. Among them, molecular descriptors, fingerprints, SMILES, and graphs are the most commonly used representations of chemical structures for organic molecules.

Molecular descriptors are either experimentally measured or theoretically derived properties of molecules [144], which have long been adopted for developing QSAR/QSPR [145,146,147]. A molecular descriptor transforms the information of a chemical structure into a mathematical form, which makes it easy to develop a quantitative relationship between structures and corresponding properties [148,149]. There are many types of molecular descriptors, such as 3D types [150,151] and topological types [152]. Some tools can be used to generate molecular descriptors, for example, the RDKit package [86], Dragon [153,154], etc.

Fingerprint, a special molecular descriptor, uses a vectored form to represent a chemical structure. Two- and three-dimensional fingerprints have been widely adopted to represent small molecules by bit strings for evaluation of compound similarity [155]. There are several classes of fingerprints, such as topological fingerprints, structural fingerprints, circular fingerprints (e.g., extended connectivity fingerprints, ECFP [88]), pharmacophore fingerprints, and hybrid fingerprints [155]. Fingerprints can be easily applied for inverse materials design. To illustrate, Huan et al. [143] adopted motif-based fingerprints to describe structures composed of C, H, O, N, and F and obtained the fingerprints-properties relationship by ML models. With this mapping at hand, they realized fast materials design by reconstructing fingerprints with target properties. However, there are limitations associated with this representation approach. For example, though it can express the presence of an element and how many of them are presented by fingerprints, it lacks the stacking information [9].

SMILES represents a molecular structure by text string, which is probably the most popular representation method. For demonstration, the SMILES form of benzene ring is C1=CC=CC=C1. However, different SMILES forms may represent the same molecule [60]. Additionally, it can not represent metallic, periodic materials, and properties that are three-dimensional geometry-dependent [156]. Furthermore, a high percentage of invalid candidates may be generated when implemented in molecular generation [60]. Nonetheless, successful applications can still be achieved based on this representation. For example, Ikebata et al. [111] developed a chemical language model following SMILES notation to generate chemically favorable molecules in a Bayesian molecular design method. The model obtained good performance of design of small organic molecules with desired internal energy and HOMO-LUMO gap.

Graph-based representation is deemed a promising method to take care of the shortcomings associated with SMILES representation [157]. It has been successfully applied to generate small molecular graphs [158,159]. In a molecular graph, atoms and bonds are represented by nodes and edges. Among many of the ML models, the VAE model is usually combined with molecular graph representation for molecular design through an encode-decode process of graphs [158,160]. Recently, Li et al. [157] proposed a conditional graph generative model which incorporated DNNs to resolve the sequential design of a molecular graph. They showed that the model built outperformed SMILES-based representation on the multi-objective (drug likeness, synthetic accessibility, etc.) drug design problem.

Feature representation can significantly affect the performance of an ML model [7]. It is reported that fingerprints are suitable for DNNs, while molecular graphs are more favorable for CNNs and RNNs [61]. It is also shown from the energetic materials design case that the sum over bond representation performs better than the custom descriptor set, Coulomb matrix, and bag of bonds representation [91,94].

#### 3.2.2. ML Methods and Model Validation

Since material properties are always assessed in the study of materials science, supervised learning methods are commonly used to learn the QSAR/QSPR from the samples that are labeled with assessed property values. Several popular ML models and respective features are briefly reviewed here. More details can be found in several recent review articles on ML methods for materials science [21,56,57,58,190].

*Linear regression* is a statistical method to regress a target or response variable on a set of explanatory variables or features. It associates a weight with each feature and sums them up to predict the response. This gives rise to a linear function in terms of the weights. Linear regression commonly minimizes the squared sum, i.e., sum of the squared error on each observed example, which leads to the least squares method. This method, although simple, is often the baseline choice for a problem.

*Gaussian process regression (GPR)* is a statistical model where observations occur in a continuous domain, such as time or space. In a Gaussian process, every point in a continuous input space is associated with a normally distributed random variable. Inference of continuous values with a Gaussian process prior is known as Gaussian process regression. Gaussian process regression is a non-parametric Bayesian approach towards regression problems. It can capture a wide variety of relations between inputs and outputs by utilizing a theoretically infinite number of parameters and determine the level of complexity from data through the means of Bayesian inference.

*Decision tree (DT)* consists of a tree structure of decisions and their possible consequences, including chance event outcomes, resource costs, and utility. It consists of nodes representing attributes, edges to branch by values of a selected attribute represented by the node from which the edge comes, and leaf nodes corresponding to the class labels or the response values. Constructing a decision tree is a top-down building procedure. It starts from a root node with the entire training data. Then in each decision node, it finds the best test attribute. By splitting the training data according to a value on this attribute, it diminishes the mixture of classes between the split sets as much as possible. To classify a test example, we start from the root, evaluate the relative test attribute, and then take the corresponding branch. This process is repeated until a leaf is encountered. The new example is then classified to the class that the leaf is labeled.

*k-nearest neighbors (kNNs)* are used for evaluation of continuous data labels and have been widely adopted for regression and classification problems. The property value is obtained by averaging over the number of *k* nearest neighbors, where *k* is an integer number specified by the users. The weight associated with each nearest neighbor can be equally contributed from its *k* nearest neighbors or assigned with different values considering their relative distances. For example, the nearest neighbor has higher weight than more distant neighbors. As such, performance of kNNs are greatly related to the local structures of the data.

*Support vector machines (SVMs)* are supervised learning methods for classification and regression analysis. SVM minimizes a loss function together with a regularizer for the best classifier (or regression model). The regularizer helps SVM find the classifier reaching the largest margin between the different classes of examples, or helps SVM penalize the models that use a lot of predictors or input variables to find sparse models. The reason that SVMs lead to superior performance is because they are able to construct a non-linear model via a linear mechanism by applying the so-called “kernel” mapping. In other words, using kernel calculation, an SVM maps the data from the input space to a high-dimensional feature space that has nonlinear terms of the input variables as coordinates first, then builds a linear model in this feature space.

*Random forest (RF)* is an ensemble learning method that predicts class labels or a response by constructing a multitude of decision trees during training. For classification, it determines the label of a new example by evaluating the mode of the classes of the individual trees. For regression, it predicts the output value of an example by averaging the values from individual trees. These decision trees are trained by a statistical method called “bagging” (which stands for bootstrap aggregating) that has been proved to reduce the model variance; hence, RF is usually not expected to overfit data. These trees differ from the standard decision trees because each node is divided using the best combination of input variables. As the trees grow, an extra randomness is brought into the process to re-shuffle a subset of features from which RF searches for the best combined feature. RF tends to give high accuracy performance in practice.

*Artificial neural networks (ANNs)* are networks connected by layers of artificial neurons which mimic the human brain. A single neuron outputs weighted inputs through a so-called activation function. A typical ANN is consisted of one input layer, an output layer, and one or more intermediate layers called hidden layers. Deep neural networks (DNNs) are special ANNs with more than one hidden layers which have superior learning power. Using nonlinear activation functions, ANNs or DNNs demonstrate excellent capability in solving highly nonlinear problems.

*Convolutional neural networks (CNNs)* are a typical deep learning method. Unlike the multi-layer perceptron (MLP, a type of feed forward neural network that consists of fully connected layers), CNNs take a multi-channeled volume as the input, such as an RGB image with three channels for the red, green, and blue color elements. In a convolutional layer, which is the main building block of CNNs, there are a set of independent filters which are also multi-channeled weights of small size. Each filter is independently convolved with the image in a manner that slides the filter over the complete image; along the way, it computes the dot product between the filter and the image patch in the sliding window. The convolved features are then passed through an activation function and a pooling layer. In a pooling layer, such as the max pooling, the maximum value from the convolved features is returned. The pooling layers help reduce the spatial size of the convolved images and extract features or representations of the image that are rotational and translational invariant. The last layers of CNNs are often the fully-connected layers to predict a response or class label based on image representation produced by the convolutional layers.

*Generative adversarial networks (GANs)* are a family of generative models that use deep neural networks. Unlike a discriminative model that is concerned with how to map from inputs to a response label, a generative model is concerned with how the input data can be generated given the label, so it often learns the distribution of the inputs. A GAN consists of two neural networks, a generator and a discriminator, that play adversarial game between each other. The generator attempts to generate new and synthetic data instances, e.g., images (after training based on a set of observed examples), whereas the discriminator evaluates them for authenticity. The goal of the generator is to generate fake images without being caught by the discriminator. The goal of the discriminator is to identify images created by the generator as fake. Hence, training a GAN requires to optimize a minimax objective function that comprises the two opposing losses. GANs have gained a lot attention lately because of their huge application potential in science, video games, fashion, art, and advertising.

A validated ML model is the foundation for accurate material property prediction and inverse materials design. However, a major issue in developing such an ML model is overfitting. In order to construct a sound ML model, *n*-fold cross-validation is usually adopted in which the dataset is split into *n* folds and *n* rounds of building ML models are carried out. In each experiment, a distinct fold is selected asthe test dataset, while the left folds are the training dataset, and the overall predictive performance of the ML model is evaluated by combining the validation results from all *n* ML models.

**Figure 10:**
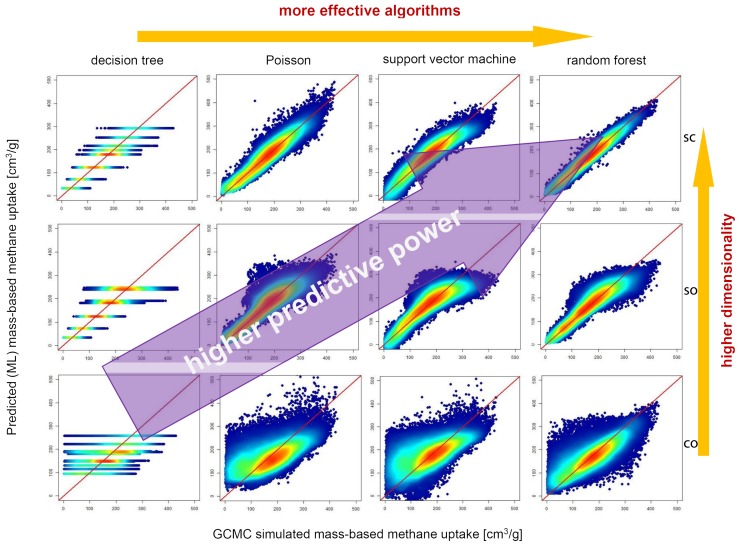
The choice of ML models and descriptors that leads to different performance of ML predictions on methane uptake of metal-organic frameworks (MOFs). Reprinted with permission from Reference [191], copyright 2017 American Chemical Society.

In summary, feature representations and ML models are two key ingredients in developing predictive ML models. Not only is individual selection of feature representation and ML model important on the ultimate performance of the ML model, their combined effect is also worthy of notice. For example, Pardakhti et al. considered the methane uptake of metal-organic frameworks (MOFs) to test the predictive capability of different ML models [191]. The dataset is taken from the database of hypothetical MOFs (hMOFs) [192], from which 130,398 candidates were extracted. Both volumetric-based uptake and mass-based uptake of methane were available in the database. Four different ML algorithms were adopted, namely decision tree (DT), Poisson regression (PR), support vector machine (SVM), and random forest (RF). To evaluate the influence of different descriptors on predictive capabilities of these ML models, the structural only (SO), chemical only (CO), and structural and chemical (SC) variables were tested to make a comparison. The structural descriptor took into account of void fraction, surface area, density, dominant pore diameter, maximum pore diameter, interpenetration capacity, and the number of interpenetration framework, while the chemical descriptor considered the types and the number of atoms, degree of unsaturation, metallic percentage, oxygen to metal ratio, electronegative atoms to total atoms ratio, and weighted electronegativity per atom. The comparison of different combinations of ML models and descriptors is shown in Figure 10. We can see that the RF model gives the best performance among these ML models, with SC descriptors.

### 3.3. Molecular Generation

**Figure 11:**
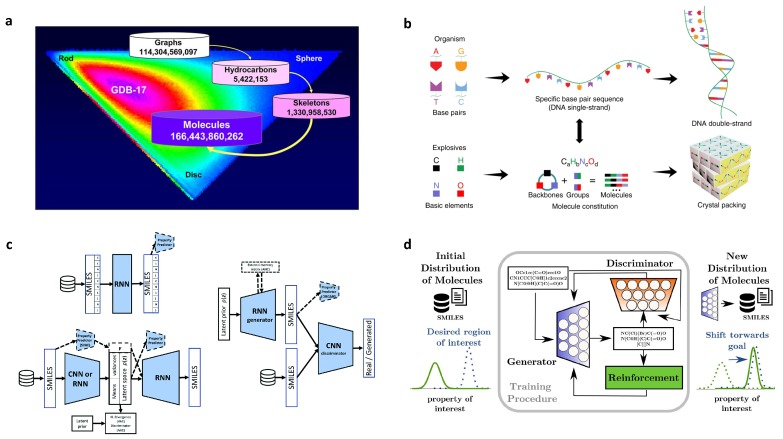
Typical molecular generation methods. (**a**) GDB molecular database generated by direct enumeration (adapted from Reference [23] with permission, copyright 2012 American Chemical Society); (**b**) high-energetic molecules generated by a material genome approach (adapted from Reference [52] with permission, copyright 2018 Springer Nature); (**c**) molecular generation by CNNs or RNNs with SMILES representation (adapted from Reference [60] with permission, copyright 2019 Royal Society of Chemistry); (**d**) molecular generation by generative adversarial network (GANs) using SMILES representation (adapted from Reference [25] with permission).

Molecular generation plays a key role in the inverse materials design process. For example, with a high-throughput screening method, molecules must be generated to form a virtual molecular library. There are different ways for molecular generation. The easiest and most direct way to generate organic molecules is perhaps by enumeration or combinatorial approaches. Taking enumeration as an example, by usage of up to 11 atoms of C, N, O, and F (saturated with H), GDB-11 database of 26.4 million molecules has been constructed [193]. Using the same fashion, GDB-13 database of 970 million molecules [108], and GDB-17 database of 166 billion molecules [23] have also been generated, as shown in Figure 11a.

The molecular generation process, in the case of GDB-17 database, starts from mathematical graphs generated by the GENG [194] program. Using up to 17 nodes by GENG and PLANARG checker of planarity of the graphs to avoid crossed bonds, 114 billion graphs is created initially. The graphs are then subjected to removal of ring strain and small rings. Consequently, 5.4 million hydrocarbons (nodes stand for C and edges denote single bond) are selected. Afterwards, the 5.4 million candidates are transferred into 1.3 billion “skeletons” by selectively substituting bond orders (single, double, and triple bonds) for the graph edges. Similarly, combinatorially substituting N and O for graph nodes, (C) is also employed. Following post-processing for oximes, nitro, S, CF_3_, and halogens, the GDB-17 database is finally generated. To verify the uniqueness of the generated molecules, the database is compared with the public archives of PubChem [195], ChEMBL [196], and DrugBank [197] in terms of molecules with up to 17 atoms. It has been found that the number of molecules in the GDB-17 database is much bigger than the total number of molecules from these three databases. Another finding is GDB-17 molecules include more rings, especially small rings and nonaromatic heterocycles. Last but no least, it contains enormous isomers of known drugs and represents various scaffold types [23].

In addition to combinations of single atoms, building fragments can also be used for combinatorial molecular generation. As mentioned in the case study of polymer dielectrics, seven building blocks were used for combinatorial generation of monomers [9]. Additionally, in the case study of polyimides design with high RIs, two types of building fragments were adopted to generate PIs [100,102]. The idea is that some building units or functional groups characterize main features of specific materials with desired properties. Thus, using these building blocks for materials design is a fast approach. Another example is the material genome approach for energetic materials design [52].C, H, O, and N were selected as the basic elements to generate small molecules mimicking the A, G, C, and T base pairs that generate DNA molecules, which are illustrated in Figure 11b. In this work, 1 parent aromatic ring from 14 candidates and certain numbers of substituent groups (each group can have 0∼3 counts) from three typical groups are chosen to combinatorially generate the organic molecules for energetic materials. With this approach, a promising candidate was successfully discovered with high energy density, as well as good thermal and mechanical stability to external stimulus, which are comparable to the most popular explosives.

Though combinatorial molecular generation methods have proven successful in real applications, they suffer from generating chemically unfavorable molecules. Therefore, much more effort is needed to virtually screen the database. Additionally, for a specific materials design task, only molecules with desired properties are needed. The molecules may only be a small portion of the whole chemical space, resulting in low efficiency of the generation effort. Moreover, combinatorial methods using known building blocks are experience biased, which significantly limits the novelty of the generated molecules [23]. To solve these problems, chemical constraints are usually applied during the generation process. These chemical constraints, such as valency rules, functional group stability criteria [23], balance of ring opening and closure, synthetic accessibility [116], and medicinal chemistry [198], can be imposed to generate chemically meaningful molecules. However, new issues still remain. On one hand, if too many constraints are imposed during the molecular generation, it is subjected to loss of novelty [116]. On the other hand, if the constraints are not enough, loss of validity of the chemical library will weaken the capability of the method [89]. It is challenging to balance these two factors for molecular generation.

Recently, deep neural networks (DNNs)-based methods have emerged to handle these problems. They have great advantages in learning the molecular grammar for representing molecules, such as SMILES, from molecule databases and creating molecules based on the information learned. There are several popular methods, such as recurrent neural networks (RNNs), autoencoder based method, generative adversarial networks (GANs), and reinforcement learning (RL). Several recent review articles provide details of these molecular generation methods [24,60,93]. Here, we briefly discuss these methods and highlight their features.

RNNs are the most popular models for sequence modeling and generation, thus having emerged as powerful generative models in different domains, such as natural language processing, music generation, and speech. Segler et al. [112] treated the task of generating SMILES as a language model attempting to learn the statistical structure of SMILES syntax by training an RNN using a large corpus of SMILES. The model can then create large sets of novel molecules with similar physicochemical properties to the molecules in the training set. Gupta et al. [199] trained a long short-term memory (LSTM)-based RNN model to generate libraries of valid SMILES strings and used a common ML strategy—transfer learning— to fine-tune a model previously trained on other sequence data. The resultant model generated molecules that were structurally similar to drugs with known activities against specific targets. This approach was successful for “low data” situations in early stage molecular discovery.

Figure 11c demonstrates the usage of RNNs or CNNs for molecular generation by learning the underlying pattern of valid strings of real molecules. Although these deep learning models could not reach 100% accuracy of finding the molecules with desired properties, the generated molecules can reach a high percentage of chemically valid species [116].

An autoencoder is a neural network that is trained to attempt to encode an input variable into latent variables (in the so-called representation or code layer) and then decode the latent variables to reproduce the input information. Variational autoencoder (VAE) is a framework for training two neural networks—an encoder and a decoder—in a Bayesian way to learn a good representation of an input structure, such as SMILES, in the code layer. Gomez-Bombarelli et al. [54] proposed a generation model using VAE to generate chemical structures after training the VAE on a large number of SMILES. The resultant latent space became a generative model. Sampling on the latent vectors and running through the decoder yield new SMILES.

SMILES strings do not directly reflect the structural similarities between molecules. Moreover, a molecule may have multiple SMILES representations, though canonization algorithms exist [200]. As a consequence, the generated molecules lack diversity and validity. Simonovsky et al. [158] proposed the GraphVAE model, which was formulated in the framework of VAE to generate molecular graphs for small molecules by predicting adjacency matrices of molecular graphs.

Some methods only generate molecules that have the same number of atoms because they can be trained using such a dataset. For instance, the GraphVAE was trained on graph data, where each graph example represented a molecule with nodes corresponding to atoms and edges reflecting the bond between any two atoms. The VAE model learns an adjacency matrix of fixed size to characterize the connectivities among the atoms, so all the training molecules had the same number of atoms. In reality, molecules that have similar structural-activity properties can have a different number of atoms and bonds. Samanta et al. [201] developed NeVAE, another deep generative model for molecular graphs, and this model allowed for graphs with different numbers of nodes and edges.

GANs are deep generative models composed of two networks: a generator and a discriminator. The generative network generates candidates, whereas the discriminator network evaluates them against the truly observed molecules. Cao et al. [159] proposed the molecular GAN (MolGAN) model, which was the first to address the generation of graph-structured data in the context of molecular synthesis using GANs. The model combined a reinforcement learning (RL) objective to encourage the generation of molecules with specific desired chemical properties by providing a reward to the created molecules in a real-time fashion during an iterative process. The limitation of MolGAN was the susceptibility to mode–collapse: both the GAN and the RL objective did not encourage the diversity of created molecules, thus the model’s tendency to be pulled towards a solution that only involves little sample variability.

Adversarial autoencoders use the GAN framework as a variational inference algorithm for both discrete and continuous latent variables in probabilistic autoencoders. Kadurin et al. [114] proposed a deep adversarial autoencoder model for identification and generation of new compounds that made a use of available biological and chemical data. The model used the NCI-60 cell line assay data for 6252 compounds profiled on MCF-7 cell line. The model output was used to screen 72 million compounds in PubChem and selected candidate molecules with potential anticancer properties. A successful example using GANs for molecular generation is the ORGANIC framework, as shown in Figure 11d, which aims to utilize GANs for molecular generation and RL for biasing lead candidates [25].

RL learns essential rules of a system by interacting with the environment, where the system resides. It learns how to take suitable actions for a given system state to maximize the expected reward in the particular situation. As is shown in the de novo drug design case, Popova et al. [116] applied RL techniques on top of a string generator to generate the SMILES strings of molecules. They successfully generated molecules with given desirable properties, though they struggled with chemical validity. You et al. [202] proposed a graph convolutional policy network (GCPN) for molecular graph generation through RL. The model was trained to optimize domain-specific rewards and adversarial loss through policy gradient, and act in an environment that incorporated domain-specific rules. The resultant model achieved 61% improvement on chemical property optimization over state-of-the-art baselines (such as GraphVAE and JT-VAE). Zhavoronkov et al. [203] developed a deep generative model, generative tensorial RL (GNETRL), for de novo small molecule design. They used GENTRL to discover potent inhibitors of discoid in domain receptor 1 (DDR1). Four compounds were active in biochemical assays, and two were validated in cell-based assays. A lead candidate was tested and demonstrated favorable pharmacokinetics in mice.

### 3.4. Inverse Materials Design

There are several levels of inverse materials design by ML models. The first level is to use trained ML model as a screening tool for high-throughput screening. In this design method, molecular generation and candidate screening are two major steps. In the second level, the two steps are combined into one cycle using RL, which biases the integrated model toward generation of valid molecules with desired properties directly. As an active learning strategy, Bayesian optimization (BO) [204] could enable an adaptive model when searching the whole chemical space with multiple objectives [24], which is an example of the third level.

#### 3.4.1. Materials Design by High-Throughput Screening

**Figure 12:**
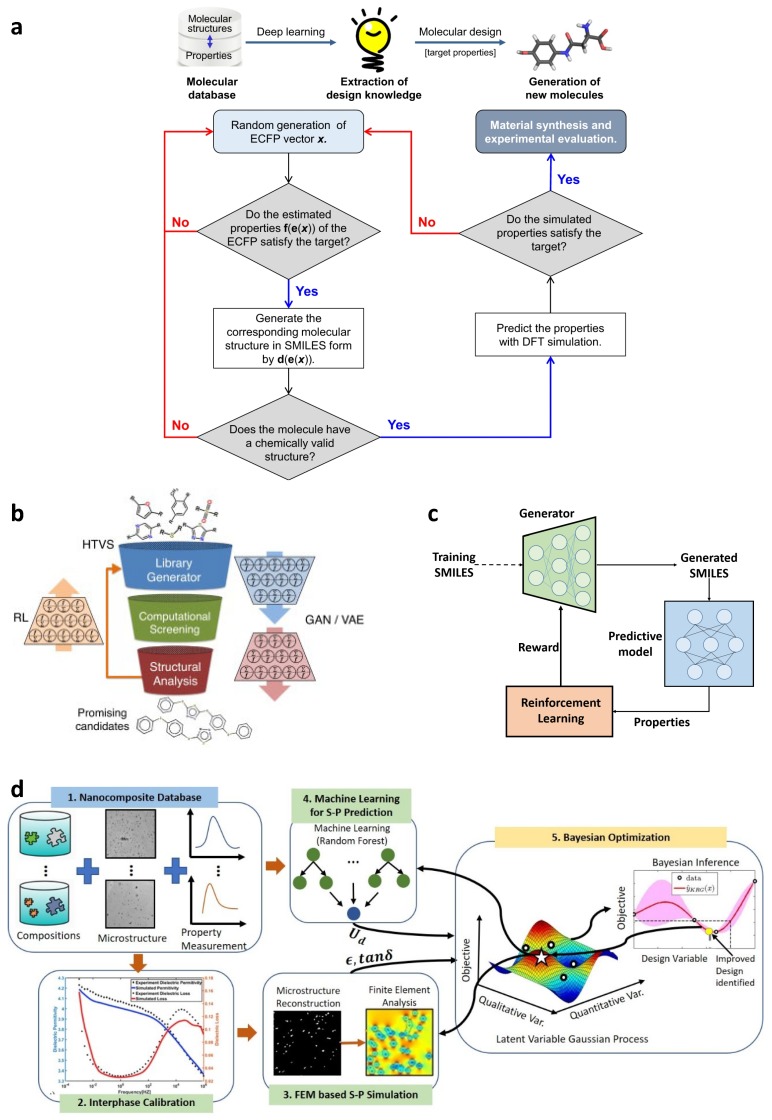
Illustration of materials design approaches. (**a**) ML-assisted materials screening (adapted from Reference [205] with permission, copyright 2018 Springer Nature); (**b**) high-throughput virtual screening integrated with ML models (adapted from Reference [206] with permission, copyright 2019 Elsevier) and (**c**) inverse molecular design by RL; (**d**) integration of various modules for design of insulating nanocomposites by Bayesian optimization (BO). ECFP = extended connectivity fingerprints.

In materials design by high-throughput screening, an ML model mapping molecular structures to their properties is established as a screening tool to filter the molecular library. An example of this design process is demonstrated in Figure 12a. In this framework, a predictive ML model is constructed first, which is then used to screen molecules generated by ML model using ECFP featurization by discriminating whether the generated molecules have the desired properties. It is supplemented by other screening constraints until it passes validation by synthesis and experimental evaluation.

However, this protocol suffers from several shortcomings. First, the candidate molecules are generated randomly without sufficiently learning the underlying grammar and rules of chemically valid structures, which could result in a high percentage of invalid molecules. Thus, it has to determine the legitimacy of generated molecules one by one, which would take enormous additional efforts. Additionally, the way of randomly generating molecules may not search through the whole chemical space uniformly and efficiently. Besides, virtual screening by DFT calculation is introduced which contradicts with the starting point of using ML models to avoid time-consuming modeling and simulation, and eventually to realize accelerated materials design. Last but not least, the development of predictive ML models for screening is separated from molecular generation, which is not an efficient approach since the percentage of desired molecules in the whole chemical space is low.

#### 3.4.2. Reinforcement Learning for Materials Design

In the second level of materials design, molecular generation is combined with molecular discrimination using RL which allows for accelerated materials design, as illustrated in Figure 12b,c. A generative model, by using either molecular graph or SMILES representation, is trained to learn the grammar of valid molecules, which can be realized by the GAN or VAE model. And then the generated molecules are judged by the predictive ML model. The RL model decides to give a reward or a penalty based on the properties predicted. Using RL, it tends to maximize the rewards, which finally biases the molecular generation process towards the space of molecules with target properties. By using such a data-driven method that attempts to learn the underlying probability distribution over a large set of chemical structures, the search over the chemical space can be reduced to only molecules seen as reasonable, without introducing the rigidity of rule based approaches.

However, this protocol suffers from a few drawbacks. First of all, when using SMILES representation, the rewards or penalties can only be given after the sequence of SMILES is generated [24], which does not allow timely feedback to generate valid SMILES. Furthermore, the use of SMILES sequence affects the capability of RL since a syntactically invalid SMILES sequence may represent a valid chemical structure [24]. Lastly, designing the reward functions is difficult when many objectives are presented, i.e., multiple properties are to be optimized. For multi-variable optimization problems, Bayesian optimization is a favorable method [207,208].

#### 3.4.3. Bayesian Optimization for Materials Design

Bayesian optimization (BO), an adaptive sampling approach driven by ML model and uncertainty quantification (UQ), has recently received significant interest in materials design. Among the many global optimization methods reported in literature, BO stands out due to its capability of locating the global optima for highly non-linear functions within tens of objective function (i.e., material properties) evaluations. BO accomplishes this by repeating these three steps [207,208]:

1. An ML model is trained on available data to predict material property of interest from the design variables and supply uncertainty quantification over the design space.
2. An acquisition function uses the prediction and UQ to determine the best design to evaluate next.
3. The design recommended by acquisition function is evaluated and added to the dataset.

This procedure is usually terminated after user-specified maximum iterations are completed. Gaussian Process modeling [209,210] is a popular choice of ML model for BO due to its inherent ability for UQ without incurring additional computational cost, although RF [211] and ensembles of SVM [212] have also been used in the past. The acquisition function essentially gauges the benefit of evaluating a design by interrogating the ML model predictions and associated uncertainties. The acquisition function must decide between exploration and exploitation of design space, which may be contradictory goals. The best performing acquisition functions generally strike a balance between the two. Commonly used acquisition functions are expected improvement [213], Probability of Improvement [214], and knowledge gradient [215]. The review article by Shahriari et al. [216] provides detailed discussions about ML models and acquisitions functions used in BO.

BO enables significant acceleration of materials design and discovery, as demonstrated by its successful application across a wide variety of materials covering alloys [217], polymers [208,218], inorganic compounds [219,220,221], and drug-like molecules [222,223,224]. A recent article by Lookman et al. [225] reviews application of BO in materials science and highlights existing challenges.

Here, we use the polymer nanocomposite as an example to demonstrate and discuss the BO for inverse materials design. Polymer nanocomposites are attractive candidates for electrical insulation [226,227]. The three key electrical properties of interest for this application—breakdown strength ($U_{d}$), dielectric permittivity (ε) and loss (tanδ)—can be tuned by careful design of constituents, their composition and nanocomposite morphology. This was demonstrated by Iyer et al. [208] using the aforementioned BO approach, with a framework that integrates experimental data in different modules of design process.

Their search space consisted of two polymers (polystyrene and polymethylmethacrylate), silica nanoparticles with three distinct monofunctional silane coupling agents (octyldimethylmethoxysilane, chloropropyledimethylethoxysilane, aminopropyledimethylethoxysilane), and infinitely many possibilities of nanocomposite microstructure. The salient microstructural features are captured by nanoparticle volume fraction (composition descriptor) and the scale parameter (dispersion descriptor) obtained from Spectral Density Function (SDF) [134,228]. The ability of represent microstructures using only a few variables is a critical aspect in materials design and has received significant interests in the last few decades. Bostanabad et al. [119] reviewed the prevalent techniques in this area and discuss their applicability.

Figure 12d illustrates the flow of information between state-of-the-art computational and experimental techniques leveraged in this design process. It commences by preparing a database (Module 1) containing TEM images and measured dielectric properties for the aforementioned polymer–surface coupling agent combinations. All TEM images were binarized using Niblack algorithm [229] and analyzed to evaluate nanoparticle volume fraction and dispersion. This process helps determine the bounds for the two microstructural design variables. The measured dielectric permittivity and loss are used to calibrate interphase dielectric properties (Module 2) for each polymer-surface coupling agent combination, through the procedure described in Wang et al. [230]. The calibrated interphase parameters are then used in a finite element program [231] (Module 3) to evaluate ε, tanδ for microstructures arising in the iterative BO loop. Evaluating breakdown strength is computationally expensive and circumvented by training a Random Forrest model on experimental data to predict $U_{d}$ from design variables.

Due to presence of qualitative (choice of polymer, surface coupling) and quantitative (nanoparticle volume fraction and scale parameter) design variables, latent variable Gaussian process [232]—a novel ML method well suited for mixed variable BO—is employed. The aim is to maximize $U_{d}$ and minimize ε, tanδ for electrical insulation. When formulated as a single objective problem, results show that BO outperforms genetic algorithm [233] by consistently identifying global optimum within 100 iterations. When formulated as a multi-objective problem, multi-objective BO identifies several designs on the Pareto frontier. All Pareto designs comprise polystyrene, clearly indicating its favorability over polymethylmethacrylate for electrical insulation. Additionally, high (low) filler volume fraction and dispersion leads to high (low) tanδ, ε and $U_{d}$. The designer may choose any Pareto design as the solution based on his/her preferences.

## 4. Conclusions

Machine learning, especially deep learning techniques, have emerged as a new and powerful method to accelerate materials design in various fields. The growth of materials databases enclosing chemical structures and corresponding properties provides huge potential to use ML-guided methods for design of organic molecules and polymers. The adequacy of data ensures that ML models sufficiently learn the underlying mapping between structures and properties, which guarantees the accuracy of QSPR/QSAR for forward property prediction. Additionally, ML models can also be used for inverse materials design in an accelerated manner. Since ML models are able to learn the syntax of chemically meaningful representations of molecules, they can be used to generate novel molecules in a more efficient way than the classical methods. Thus, it has many advantages to deal with issues associated with inverse materials design for organic molecules and polymers.

In this work, we have reviewed recent progress using ML-guided materials design in chemical, biomedical, and materials science fields. Nine representative design examples are highlighted and examined. More importantly, as scale of materials design influences the ultimate performance of the materials, both molecular and microstructure designs of OPVs are presented and discussed. Challenges and issues associated with ML models to tackle materials design problems are discussed. Specifically, the challenges are as follows:

(i) *Acquisition of a diverse database.* There are many public databases available for various materials, such as the ones summarized in Table 2. If no database of interest is available, we can build one by experiments or simulations. As a result, it is generally not challenging to acquire a database, rather it is challenging to obtain a “good” one. “Good” means that the database is diverse or uniform across the chemical space [140] since this feature of a database significantly affects the capabilities (interpolation, extrapolation, and exploration) of the ML model to be built. With a diverse or uniform database in the chemical space, the ML model guarantees the prediction by interpolation, while with a database in a limited region or class, the prediction is weakened by extrapolation. However, since the whole chemical space is nearly infinite and not clearly known, how can we determine if the database is uniform or not? To overcome this challenge, two areas of algorithmic approaches should be considered [140]: algorithms to perform searches, and more general machine learning and statistical modeling algorithms to predict the chemistry under investigation. The combination of these approaches should be capable of navigating and searching chemical space more efficiently, uniformly, quickly and, importantly, without bias [140].
(ii) *Feature representation.* Most ML models need all inputs and outputs to be numeric, which requires the data to be represented in a digital form. Many types of representation methods are widely used, such as molecular descriptors, SMILES, and fingerprints, as summarized in Table 3. However, are they universal for all property predictions? Taking fingerprints as an example, it is known that different functional groups (substructures) of a complex structure may have distinct influences on the properties. Therefore, if one fingerprint method with certain bits does demonstrate predictive power in one property prediction, will it have the same capability in another property prediction? In addition, which representation is more suitable to work with specific ML models so that the model can have strong predictive capability? All of these questions require us to be cautious for the feature representation, selection, and extraction by applying the ML models for different materials and properties.
(iii) *ML algorithms and training.* When conducting a materials design task, the choice of a suitable ML model should be carefully considered. There are many available ML models to choose as reviewed in the Discussion section, but it is not as easy as just to choose any one randomly. Choosing a suitable ML model depends on the database availability and the feature representation method. Which ML model is the best for a certain material property prediction? Does it depend on the type of materials? Can a model that is built with strong predictive power for one material be applicable to other similar but different materials? What about applying to a totally different material? Additionally, when training the selected ML model, there are usually some hyperparameters to be set. It is not trivial to set them without any knowledge of the ML algorithms. In order for the ML model to have better predictive power, the setting of these hyperparameters needs learning efforts, from the user’s point of view.
(iv) *Interpretation of results.* ML models do show good prediction power in some cases. However, how to explain the constructed model, for example, the DNN model, is still an open question even in the field of computer science. When applying ML models to materials design, is there any unified theory to physically or chemically interpret the relationship established between a chemical structure to its properties? Can the model built increase our understanding of materials? What role should we consider ML models to be in materials design?
(v) *Molecular generation.* Molecular generation plays an important role in the design of de novo organic molecules and polymers. As we have discussed, there are several deep generative models, including generative adversarial networks, variational autoencoders, and autoregressive models, rapidly growing for the discovery of new organic molecules and materials [24,60,93]. It is very important to benchmark these different deep generative models for their efficiency and accuracy. Very recently, Zhavoronkov and co-workers have proposed MOlecular SEtS (MOSES) as a platform to benchmark different ML techniques for drug discovery [234]. Such a platform is extremely helpful and useful to standardize the research on the molecular generation and facilitate the sharing and comparison of new ML models. Therefore, more efforts are needed to further design and maintain these benchmark platforms for organic molecules and polymers.
(vi) *Inverse molecular/materials design.* Currently, RL has been widely used for the inverse molecular/materials design, due to its ease of integration with deep generative ML models [25,36,116]. RL usually involves the analysis of possible actions and outcomes, as well as estimation of the statistical relationship between these actions and possible outcomes. By defining the policy or reward function, the RL can be used to bias the generation of organic molecules towards most desirable domain [24,25,116]. Nevertheless, the inverse design of new molecules and materials typically requires multi-objective optimization of several target properties concurrently. For instance, drug-like molecules should be optimized with respect to potency, selectivity, solubility, and drug-likeness properties for drug discovery [116]. Such a multi-objective optimization problem poses significant challenges for the RL technique [235,236,237], combined with the huge design space of organic molecules. Comparing with RL technique, BO is more suitable and effective for multi-objective optimization and multi-point search [238,239,240]. Yet, the design of new molecules and materials involve both continuous/discretized and qualitative/quantitative design variables, representing molecular constituents, material compositions, microstructure morphology, and processing conditions. For these mixed variable design optimization problems, the existing BO approaches are usually restrictive theoretically and fail to capture complex correlations between input variable and output properties [207,208,232]. Therefore, new RL or BO methods should be formulated and developed to resolve these issues.

Nevertheless, with these successful design examples summarized in Table 1, we are positive that the gap between the promise of ML-assisted materials design approaches and their broad applications in reality will be minimized. We anticipate that ML-assisted materials design for organic molecules and polymers will be the driving force in the near future, when accelerated materials design is fully realized to meet the tremendous demand of new materials with tailored properties in different fields.

## Figures and Tables

**Table 1:** Summary of the nine ML-guided materials design examples.

Materials	Design Feature	Design Scope	Data Size	Representation	ML Model
Organic photovoltaics (2011)	Self-built library and screening	Power conversion efficiency (molecular level)	2.6M	Molecular descriptors	MLR
Polymer dielectrics	Self-build library; building blocks for molecular generation; genetic algorithm	bandgap and dielectric constant (molecular level)	284	Fingerprints	KRR
Organic light-emitting diodes	Self-build library and screening; building blocks for molecular generation	delayed fluorescent rate constant (molecular level)	40,000	ECFPs	ANN
Polymer solar cell (2018)	Self-build library and screening; building blocks for molecular generation; various combinations of feature representations and ML models are compared	highest occupied molecular orbital (HOMO) and lowest unoccupied molecular orbital (LUMO) (molecular level)	3938	Fixed length vector; string; spatial coordinate	LRR; MLP; RF; DTNN; GrammarVAE
High-energetic material	Material design with limited data; various combinations of feature representations and ML models are compared	high energy density and low sensitivity (molecular level)	109; 309	CDS; SoB; CM; BoB; fingerprints	KRR; RR; SVR; RF; kNN; LASSO; GPR; ANN
Polyimides with high refractive index	Self-build library and screening; building blocks for molecular generation; ML model construction with limited data	polarizability and number density (molecular level)	196	Number of monomer units	SVM
Polymer with high thermal conductivity	ML model construction with limited data; transfer learning	thermal conductivity (molecular level)	28; 5917; 3234	ECFPs	Bayesian model
de novo drug-like molecule	Material design with arbitrary target property range; SMILES strings as input for molecular generation	physical/chemical/biological properties (molecular level)	1.5M	SMILES	DNN; RL
Organic photovoltaic solar cells (2019)	Polymer composite design; bottom-up nanofabrication; microstructure characterization and reconstruction	IPCEefficiency (microstructure level)	45	Microstructure characterization	SDF

Note: ECFPs: extended-connectivity fingerprints; CDS: custom descriptor set; SoB: sum over bonds; CM: Coulomb matrix; BoB: bag of bonds; SMILES: simplified molecular-input line-entry system. MLR: multi-linear regression; KRR: kernel ridge regression; ANN: artificial neural network; LRR: linear ridge regression; MLP: multi-layer perceptron; RF: random forest; DTNN: deep tensor neural network; GrammarVAE: grammar variational autoencoder; KRR: kernel ridge regression; RR: ridge regression; SVR: support vector regression; kNN: k-nearest neighbors; GPR: Gaussian process regression; SVM: support vector machine; DNN: deep neural network; RL: reinforcement learning; SDF: spectral density function.

**Table 2:** Some public materials databases enclosing structures and properties.

Database	Type	Description	URL
AFLOWLIB	Computation	Database of 2,961,744 material compounds with over 527,190,432 calculated properties	http://aflowlib.org
BNPAH	Computation	Structures and properties of 77 polycyclic aromatic hydrocarbons and 33,059 B, N substituted compounds	https://moldis.tifrh.res.in/datasets.html
ChemDiv	Comp./Exp.	Collection of over 1,500,000 individually crafted, lead-like, drug-like small molecules	http://www.chemdiv.com/complete-list/
ChemSpider	Experiment	A free chemical structure database providing fast text and structure search access to over 67 million structures	https://chemspider.com
ChEMBL	Experiment	A manually-curated database of bioactive molecules with drug-like properties	https://www.ebi.ac.uk/chembl
Citrination	Experiment	A premier open database and analytics platform for the world’s material and chemical information	https://citrination.com
CMR	Computation	A collection of molecules obtained from electron-structure codes	https://cmr.fysik.dtu.dk
COD	Experiment	A collection of crystal structures of organic, inorganic, metal-organics compounds, and minerals, excluding biopolymers	http://www.crystallography.net/cod/
CSD	Experiment	A database of over one million small-molecule organic and metal-organic crystal structures	https://www.ccdc.cam.ac.uk
DrugBank	Experiments	Drug database with comprehensive drug target information	https://www.drugbank.ca/
eMolecules	N/A	Commercially available with over seven million compounds for drug discovery	https://reaxys.emolecules.com/index.php
Energetics	Computation	A database of energetic molecules	https://git.io/energeticmols
GDB	Computation	A database containing hypothetical small organic molecules	http://gdb.unibe.ch/downloads
HCEP	Computation	Harvard Clean Energy project for solar absorber materials	https://cepdb.molecularspace.org
HOPV15	Comp./Exp.	A collation of experimental photovoltaic data from the literature and calibrated by DFT calculation	https://figshare.com/articles/HOPV15_Dataset/1610063/4
ICSD	Experiment	A database of inorganic crystal structure	https://icsd.fiz-karlsruhe.de
MatNavi	Experiment	A materials databases of polymer, ceramic, alloy, superconducting material, composite, and diffusion	http://mits.nims.go.jp
MatWeb	Experiment	A database of material properties of polymers, metals, ceramics, and semiconductor	http://matweb.com
MP	Computation	Computed information on known and predicted materials	https://materialsproject.org
NIST CW	Experiment	A database of thermochemical properties	https://webbook.nist.gov/chemistry
NIST MDR	Experiment	A repository of material data being updated	https://materialsdata.nist.gov
NOMAD	Computation	A repository to host, organize, and share material data	https://nomad-repository.eu
NREL MD	Computation	A computational materials database for renewable energy applications	https://materials.nrel.gov
OQMD	Computation	A database of DFT-calculated thermodynamic and structural properties	http://oqmd.org
PubChem	Experiment	A chemical database of chemical and physical properties, biological activities, and safety and toxicity information	https://pubchem.ncbi.nlm.nih.gov
QM	Computation	Small organic molecules calculated by DFT	http://quantum-machine.org/datasets/
TEDesignLab	Comp./Exp.	Thermoelectric material design	http://tedesignlab.org
ZINC	Computation	Database of commercially-available compounds for virtual screening	https://zinc15.docking.org

**Table 3:** Common feature representations of organic molecules and tools for feature generation.

**Representation**	**Description**	**References**
SMILES	Line notation for describing a chemical structure using text strings	[87,112,115,116]
Fingerprints	A special descriptor using vector of fixed or variable length to represent a chemical structure	[58,143,155,161]
Molecular graphs	A representation of chemical structures by graph theory	[157,158,159,160]
Coulomb matrix	A matrix representation embedded nuclear coordinates and charges, similar representations include Ewald sum matrix, Sine matrix	[90,91,162,163,164]
Smooth overlap of atomic orbitals (SOAP)	A special descriptor encoding atomic structures using local expansion of atomic density	[165,166,167]
Atom-centereded symmetry functions (ACSF)	A special descriptor representing the local environment near an atom using two- or three-body functions	[168,169,170]
Bag of bonds	A vector enclosing chemical bonds and corresponding numbers	[91,92]
Grids of molecules	A visual form of molecules generated by their coordinates	[61,171,172]
**Tools**	**Description**	**References**
CDK	Chemistry Development Kit: open-source Java libraries for cheminformatics to generate various descriptors, fingerprints, etc.	[173,174,175,176]
ChemDes	A free web-based tool for generation of molecular descriptors (3679 types) and fingerprints (59 types)	[144,177]
ChemMine	A free online tool for analyzing and clustering small molecules, including similarity search and properties calculations	[178,179]
OEChem	Programming library for chemistry and cheminformatics with small molecules	[180,181,182]
Open Bable/Pybel	Open-source chemical toolbox to search, convert, analyze, and store data	[183,184,185]
PaDEL	A software to generate molecular descriptors (1875 types) and fingerprints (12 types) using CDK	[186,187]
PubChemPy	An open-source python library to interact with PubChem	[188]
RDKit	A collection of cheminformatics and machine-learning tools	[86,189]
